# Rational Design and Construction of Cocatalysts for Semiconductor‐Based Photo‐Electrochemical Oxygen Evolution: A Comprehensive Review

**DOI:** 10.1002/advs.201801505

**Published:** 2018-11-19

**Authors:** Xiao‐Ting Xu, Lun Pan, Xiangwen Zhang, Li Wang, Ji‐Jun Zou

**Affiliations:** ^1^ Key Laboratory for Green Chemical Technology of the Ministry of Education School of Chemical Engineering and Technology Tianjin University Tianjin 300072 China; ^2^ Collaborative Innovative Center of Chemical Science and Engineering (Tianjin) Tianjin 300072 China

**Keywords:** cocatalysts, oxygen evolution reaction, photoanodes, solar energy, water splitting

## Abstract

Photo‐electrochemical (PEC) water splitting, as an essential and indispensable research branch of solar energy applications, has achieved increasing attention in the past decades. Between the two photoelectrodes, the photoanodes for PEC water oxidation are mostly studied for the facile selection of n‐type semiconductors. Initially, the efficiency of the PEC process is rather limited, which mainly results from the existing drawbacks of photoanodes such as instability and serious charge‐carrier recombination. To improve PEC performances, researchers gradually focus on exploring many strategies, among which engineering photoelectrodes with suitable cocatalysts is one of the most feasible and promising methods to lower reaction obstacles and boost PEC water splitting ability. Here, the basic principles, modules of the PEC system, evaluation parameters in PEC water oxidation reactions occurring on the surface of photoanodes, and the basic functions of cocatalysts on the promotion of PEC performance are demonstrated. Then, the key progress of cocatalyst design and construction applied to photoanodes for PEC oxygen evolution is emphatically introduced and the influences of different kinds of water oxidation cocatalysts are elucidated in detail. Finally, the outlook of highly active cocatalysts for the photosynthesis process is also included.

## 1 Introduction

Constant and unhealthy development of modern civilization and industrialization results in worsening a series of environmental problems. The traditional fuel resources are close to be exhausted, the ecosystem is deteriorated, and the environmental pollution is much worse than imagination. Therefore, effective solutions should be proposed to address these issues.^1^, ^2^, ^3^, ^4^, ^5^ It is said that the existence of environmental issues partly attributes to the unreasonable use of traditional fossil fuel, such as excessive exploitation and inefficient utilization. Confronted with this dilemma, it is impending to improve the efficiency of traditional resources or explore new, clean, and sustainable ones if we want to alleviate the environment pressure. Recently, mounting researchers conduct their study based on sustainable energy like solar energy, wind energy, bioenergy, etc. Among them, solar energy is vastly abundant, low cost, and carbon‐free, hence becoming dominant and emerging the potential application perspectives.^2^, ^3^, ^4^

**Figure 1:**
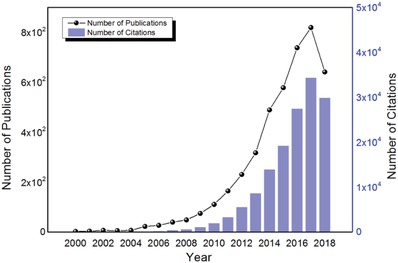
The number of publications and citations in the field of photo‐electrochemical water splitting from 2000 to 2018. During this period, these data climbed up exponentially. The raw data were collected from a literature search in the Web of Science database on 6 October 2018 with the key words “photo‐electrochemical water splitting.”

Photo‐electrochemical (PEC) water splitting is a facile photosynthesis route and defined as an electrochemical process which occurs when a semiconductor electrode is under light irradiation in order to convert sunlight into oxygen or hydrogen energy.^5^ It is cost‐competitive, free of collateral contamination, and does not require additional separation process, thus has attracted increasing attention recently. As shown in **Figure** **1**, an exponential growth of related published papers begins from 2004, and the total number of publications and citations in this field reached 4344 and 147 015, respectively (up to 6 October 2018). It indicates the PEC water splitting process becomes more and more important under the current global energy crisis and environmental pollution.

PEC water splitting was first found by Fujishima and Honda, who used TiO_2_ photoanode to split water into H_2_ and O_2_ successfully under ultraviolet (UV) light in 1972.^6^ It is considered as the remarkable milestone of solar energy utilization field and caused an explosive impact in this field. Theoretically, the limited efficiency for a single semiconductor photoelectrode with a bandgap around 1.5 eV under 1 sunlight irradiation (100 mW cm^−2^) is possible to attain ≈30%, hence it can reach a high yield of hydrogen and oxygen.^7^, ^8^ Whereas, to the best of our knowledge, the current efficiency of PEC water splitting is still much lower than the ultimate efficiency and far from meeting the demand of industrialization owing to the following shortcomings:

1) Some of the traditional photoanodes have wide bandgap and can only seize UV light, resulting in low utilization of sunlight and energy conversion efficiency.^9^ 2) The lifetime of photogenerated carriers is very short. The excited electrons are inclined to recombine with holes in order to maintain a stable state, which has been considered as a major factor of the energy loss. Therefore, after irradiation, only a small portion of the excited electrons and holes can successfully move to the different surface areas of the electrodes and participate in water splitting. 3) Many common photoanodes are electrochemically instable in electrolyte environment or easy to be corroded by photoinduced holes, which will oxidize materials instead of water and reducing the PEC water splitting performance.

Hitherto, these three major problems are still the stumbling blocks for the further development of photoelectrodes. In this case, novel surface engineering strategies have been explored constantly in order to address these problems in terms of optimizing the bandgap of photoelectrodes, restraining recombination, facilitating the immigration of charge carriers for water splitting, as well as improving the stability against photocorrosion and decomposition.^10^ These strategies mainly include morphology control, heterojunction/homojunction formation, ions doping, cocatalysts engineering, etc. For example, Wu et al.^11^ and Wang et al.^12^ demonstrated that by tuning morphology of photoanodes, the efficiency of O_2_ generation in the PEC water splitting reaction can be optimized. Su et al.^13^ and Hong et al.^14^ proposed that the heterojunction formed between interface of WO_3_ and BiVO_4_ was conducive to charge transfer and recombination suppression. Pan et al. constructed TiO_2_ p–n homojunction to realize enhanced PEC H_2_ evolution performance.^15^ Yang et al. rationally synthesized nitrogen‐doped ZnO nanowire arrays as photoanodes and the obtained photocurrent of ZnO:N increased by an order of magnitude than that of bare ZnO.^16^

Alternatively, engineering cocatalysts on the surface of photoelectrodes gradually came to sight recently, as the existence of cocatalysts can bring about the reduced overpotential, accelerated reaction kinetics, enriched active sites, and suppressed corrosion.^17^ Besides, the methods to incorporate cocatalysts with photoelectrodes such as electrodeposition or photo‐electrodeposition are facile and easy to implement. Virtually, it is revealed that most of the excellent PEC water splitting cocatalysts are electrocatalysts,^18^, ^19^, ^20^ such as transition metal dichalcogenides (TMDs),^21^, ^22^, ^23^, ^24^, ^25^ phosphorous metal compounds,^26^, ^27^, ^28^ metal oxides,^29^, ^30^, ^31^, ^32^ metal hydroxides,^29^, ^33^ etc. These electrocatalysts not only play a vital role in electrocatalysis but also have a significant impact on PEC water splitting. Therefore, in a manner of speaking, the discovery of robust electrocatalysts provides numerous possibilities for cocatalyst engineering strategy and then remarkably promotes the progress of photo‐electrocatalysis.

In general, cocatalysts consist of hydrogen evolution reaction (HER) and oxygen evolution reaction (OER) cocatalysts. Compared with HER cocatalysts, OER cocatalysts have more valuable status for PEC water splitting and are explored more extensively. The reasons are as follows: compared with the abundant variety of PEC OER cocatalysts, materials used for PEC HER are limited and most of them are relatively unstable such as TMDs.^25^ In addition, water oxidation half reaction is much more demanding than water reduction. Therefore, it is essential to improve the water oxidation capability of photoanodes for better PEC overall water splitting performance. With suitable OER cocatalysts, the photoanodes used for light‐induced water oxidation and even the entire PEC process will be highly efficient.

Some reviews related to the rational design and fabrication of photoanodes for PEC water splitting have been published, but few of them are comprehensive with respect to the construction of PEC water oxidation system and especially the functions of cocatalysts deposited onto photoanodes. Hence, there is still a large space for us to further elucidate and discuss from these aspects as well as summarize the progress of the development in a more accessible and detailed way. It needs to be emphasized that this review mainly focuses on the basic information of PEC water oxidation half reaction and the recent advances of OER cocatalysts research applied on the photoanode in order to enhance the efficiency of PEC oxygen production. In the discussion, the modules of a typical PEC cell and the basic mechanism of PEC water splitting will be simply introduced. The benchmark and the diagnostic evaluation parameters commonly used in PEC OER process and the intrinsic function of cocatalysts will be demonstrated. Different categories of water oxidation cocatalysts will be classified and compared. Meanwhile, the typical examples of cocatalysts coupled with photoanodes will be overviewed and the optimization methods to promote catalytic activity of cocatalysts will be described.

## 2 Basic Principles

### 2.1 PEC Water Splitting Process

**Figure 2:**
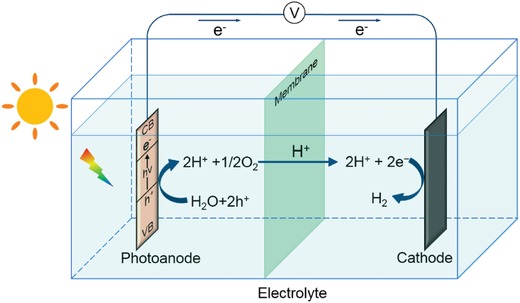
A sketch of the PEC cell for water splitting.

A single PEC water splitting cell is consisted of electrodes (at least one photoelectrode), aqueous electrolyte, as well as electric wire which connects the photoelectrodes (**Figure** **2**). The substrate of photoelectrode is responsible for the growth of semiconductor materials and transfer of electrons in the external circuit. Some common substrates include metal foils and transparent conductive oxide (TCO)‐coated glasses. The photoelectrodes work as a sunlight absorber, capturing solar energy to driving water splitting. The electrolyte is also an essential part of the system, as it assists in ionic migration in the internal circuit, and the commonly used electrolytes are Na_2_SO_4_, NaOH, K_2_SO_4_, etc. When the whole system is under sunlight irradiation, the electron from the photoanode will be excited from valence band (VB) to the unoccupied conduction band (CB) with the hole left on VB after absorbing photons, the energy of which is equal to or higher than the bandgap, as is shown in Equation (1). Then some of the excited electrons transfer over the external circuit to the interface of the cathode and electrolyte and cause the reduction of hydrogen ions, while some of the *h^+^* diffuse to the surface of photoanode and assist oxidation of water. The related half‐reaction equations on two electrodes are shown in Equations (2) and (3). In fact, it is impossible for all the electron–hole pairs to transfer to the different electrode surfaces respectively after excitation due to the recombination, thus a bias will be added to the PEC system in order to promote the migration of excited charges and suppress the opportunity of recombination.^34^ This bias can be an external voltage provided by a power source, an internal voltage provided by H^+^ of different concentrations in the electrolyte, or an internal voltage by integrating a photovoltaic cell with this system^8^

(1) $$h\gamma \to e^{-}+h^{+}$$

where *h* is the Planck's constant, γ is the frequency of light, *e^−^* is photogenerated‐electrons, and *h^+^* is photogenerated‐holes.

Photoanode

(2) $$H_{2}O+2h^{+}\to 2H^{+}+1/2O_{2}\;E_{\left(\mathrm{ox}\right)}=-1.23\;V\;\mathrm{vs}\;\mathrm{RHE}$$

Cathode

(3) $$2H^{+}+2e^{-}\to H_{2}\;E_{\left(\mathrm{red}\right)}=0\;V\;\mathrm{vs}\;\mathrm{RHE}$$

Comparing Equation (2) with (3), it can be seen that water oxidation half reaction is thermodynamically and kinetically demanding. Its energy barrier is much higher and the process is more complex than water reduction. Four holes will be involved to produce one mole of oxygen while only two electrons participate in the reaction for generating one mole hydrogen. Therefore, the water oxidation half reaction is considered as one of the major challenging steps of the PEC water splitting.

**Figure 3:**
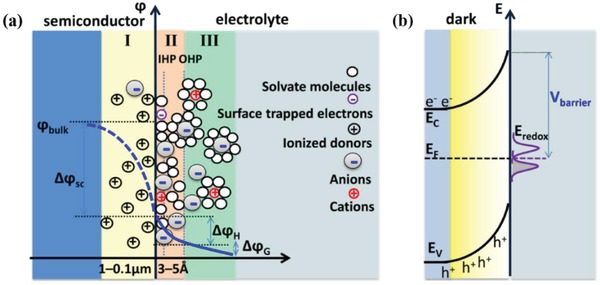
a) The structure model of the n‐type semiconductor/electrolyte interface under equilibrium conditions and the potential (ϕ) profile across this interface (blue line). Δϕ_SC_, Δϕ_H_, and Δϕ_G_ are the potential drops in the space charge layer (I), Helmholtz layer (II), and Gouy layer (III), respectively. b) The electron energy (*E*) profile of an n‐type semiconductor in equilibrium with the electrolyte in dark. *E*_C_ is the energy of the conduction band, *E*_V_ is the energy of the valence band, and *V*_barrier_ is the barrier height caused by upward band bending. Reproduced with permission.^17^ Copyright 2016, American Chemical Society.

It is worth noting that the PEC water splitting takes place at the interface of electrodes and electrolyte. Therefore, it is necessary to understand the conditions of the interface so as to have more insights into the entire reaction processes. However, due to the existence of multiple processes and the unclear reaction mechanisms at the interface, it is very difficult to make the experimental measurements. Thus, researchers turn to modeling and simulation study, which allow for rational computational design of the interface at the atomistic level.^35^ Based on these techniques, a common structure model of the semiconductor/electrolyte interface can be set up.^17^ As is seen in **Figure** **3**, three layers exist at the interface. In the near surface of the semiconductor, a space charge layer with a thickness of ≈1–0.1 µm is formed because of the nonequilibrium of the Fermi level (*E*_F_) of the semiconductor and the redox potential of the electrolyte (*E*_redox_). In most conditions, the space charge layer is a depletion layer full of immobile ionized donors, thus containing positive charges. The interlayer, named as Helmholtz layer, is a negatively charged layer from the solution side and is about 3–5 Å in thickness, consisting of trapped electrons in surface states, adsorbed ions, oriented water molecule dipoles, etc. Next to the Helmholtz layer is the Gouy diffuse layer with the lowest potential among three layers. It is filled with numerous anions and few cations. Due to the different electron densities in these three layers, a potential drop across them exists, causing upward band bending and changing the Fermi level of the semiconductor.

**Figure 4:**
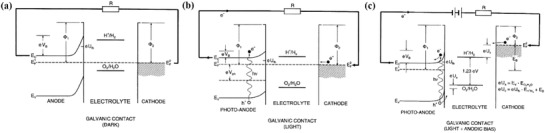
Energy diagram of PEC components: anode (n‐type semiconductor), electrolyte, and cathode (metal) with a) galvanic contact (dark), b) galvanic contact under light, and c) galvanic contact, external bias, and light, respectively . Reproduced with permission.^8^ Copyright 2002, Elsevier.

After connecting the semiconductor with the other electrode, an electrochemical chain of a PEC water splitting system can be obtained (**Figure** **4**).^8^ From the changes of their band structures, it can be seen that the entire processes can be divided into three main stages: Figure 4a shows that the galvanic contact under dark is formed after connecting the semiconductor and metal with electric wire, which results in the electronic charge transfer from the semiconductor to the electrolyte and the upward band bending due to their difference of energy levels. In this case, the Fermi level of the cathode is lower than the energy level of H^+^/H_2_, hindering the process of PEC water splitting. Once the above system is exposed under light (Figure 4b), the surface potential of the photoanode and the H^+^/H_2_ potential will be reduced. However, at this moment, the H^+^/H_2_ potential is still above the Fermi level of the cathode. In the end, an external bias is added to the circuit, causing the H^+^/H_2_ potential to be lower than Fermi level and then facilitating PEC water splitting proceeding (Figure 4c).

### 2.2 Primary Components of a PEC Water Oxidation Process

#### 2.2.1 Substrates

A substrate is the essential element of PEC anodes, because it connects the electric wires with the photoelectrode materials. For photoanodes, a conductive material with a smaller work function than that of the semiconductor is qualified to act as a substrate in order to ensure optimal charge carrier transfer at the interface between the semiconductor and the substrate.^36^ Furthermore, accessibility, economy, and flexibility are also important parameters when selecting a substrate. The typical substrates including metal substrates, metal‐free substrates, and TCO‐coated glasses were demonstrated as follows.

Metal substrates, such as Pt,^37^ Ti,^37^ Ta,^38^ copper foils,^39^ nickel foams,^40^ are usually flexible, heat resisting, and of high conductivity. Particularly, stainless steel,^41^, ^42^ which contains common transition metallic elements, like Fe, Ni, can be the source of in situ growth of the PEC catalyst, and thus becomes a suitable substrate alternative. Besides, some metal‐free materials like silicon,^43^, ^44^ carbon cloth,^45^ and ultralong carbon nanotubes (CNTs)^46^ can also be applied as substrates due to their good conductivity. Very recently, Kecsenovity et al. revealed the deposition of Cu_2_O onto different graphene substrates including 3D graphene foams, CNT networks, and spray‐coated graphene films, and found the properties of graphene foams were superior to another two substrates because of its 3D interconnected structure and high conductivity.^47^ Generally, the most extensively used substrates currently were TCO‐coated glass such as fluorine‐doped tin oxide (FTO) and indium tin oxide (ITO). These materials are affordable, corrosion‐resistant, versatile, and of excellent transparency. Though they may have a lower intrinsic conductivity in comparison with metal or carbon substrates, it can be offset by adding an ultrathin underlayer of SiO*_x_* or Nb_2_O_5_. To date, various photoanodes have been assembled based on these substrates.^48^, ^49^, ^50^ For instance, Yang et al. demonstrated the growth of ZnO:N nanowires onto ITO substrates successfully.^16^ Vertically aligned Fe_2_O_3_ nanorod arrays were also seeded onto FTO substrates uniformly by An et al.^49^

**Figure 5:**
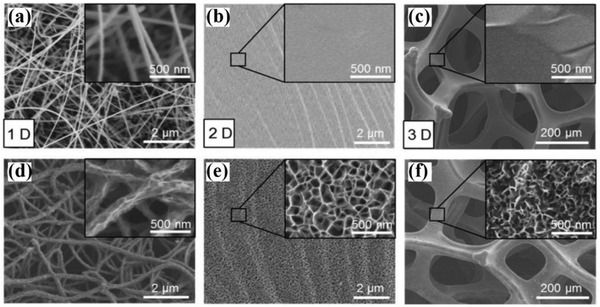
Scanning electron microscope (SEM) images of typical substrates before and after growth of GDY nanowalls: a,d) on 1D silicon nanowires, b,e) on 2D Au foil, and c,f) on 3D GF grown on Ni Foam. Reproduced with permission.^52^ Copyright 2016, Wiley‐VCH.

As the semiconductors are usually seeded onto the surface of substrates, the surface chemistry of substrates could significantly influence their growth condition and physicochemical properties.^51^ Gao et al. reported the growth of structure‐controlled graphdiyne (GDY) nanowalls on 1D, 2D, and even 3D substrates (stainless steel mesh and graphene foam (GF)), which emerge a great difference in their morphologies, chemical structures, and optical properties.^52^ The morphology before and after growing GDY is shown in **Figure** **5**.

#### 2.2.2 Electrolytes

The electrolyte of a PEC cell is another essential element affecting the PEC water oxidation. A tiny change in the electrolyte conditions may even result in a momentous variation in the interface catalysis.^53^ Good conductivity is an important principle to choose electrolytes in order to smooth charge transfer in the circuit. Employing active cations like K^+^, Na^+^, and anions like SO_4_^2−^ or OH^−^ with appropriate concentration (≈0.1 m or above) contributes to minimize solution resistance and facilitates band bending.^54^ Hence, selecting highly conductive electrolytes is a critical part of the PEC water oxidation process.

Moreover, the pH values of the electrolytes should also be taken into consideration, as the stability of the photoanodes, the redox potential, and the rate of water oxidation reaction are all highly depended on it.^55^ A reliable reference for electrolyte selection is Pourbaix diagrams, which indicates the stable phases of materials when varying different pH values.^56^ Additionally, as for oxygen evolution (Equation (2)), high concentration of H^+^ will restrain the half reaction from proceeding in the positive direction. From this perspective, low pH is not beneficial for water oxidation which can be verified by **Table** **1**. Meanwhile, a shift of the band level of a semiconductor material can also occur due to the change in pH value, which will also further affect the efficiency of PEC water oxidation.^57^ Therefore, as a trade‐off, it is necessary to find a suitable pH to satisfy above requirements simultaneously.

**Table 1:** Summary of the conversion efficiencies of photocurrent to oxygen obtained in electrolytes with varied pH conditions and anions. Reproduced with permission.^59^ Copyright 2012, American Chemical Society

Electrolytes	pH	Photocurrent to oxygen conversion efficiency [%]
CH_3_COOH	3	0
CH_3_COONa	5	0
NaCl	1	0
NaCl	3	0
NaCl	5	0
NaH_2_PO_4_	1	33
NaH_2_PO_4_	3	58
NaH_2_PO_4_	5	79
NaClO_4_	1	32
NaClO_4_	3	29
NaClO_4_	5	9
Na_2_SO_4_	1	35
Na_2_SO_4_	3	63
Na_2_SO_4_	5	88

In addition, the electrolytes need to be compatible with the given photoelectrode. They should not have chemical interactions with photoelectrodes, and their light absorption range cannot overlap with that of photoelectrodes.^58^ Versatile electrolytes that can be compatible with many photoanodes include Na_2_SO_4_, K_3_PO_4_, NaClO_4_, NaOH, etc. Table 1 shows the efficiency of PEC water oxidation on WO_3_ with different electrolyte types and pH values (at 1, 3, and 5). It is worth noting that both the electrolyte types and pH values will affect oxidation efficiency. CH_3_COOH, CH_3_COONa, and NaCl are not compatible with WO_3_, hence the photoanode cannot converse the photocurrent to oxygen when immersed in these electrolytes. Besides, as for different anions, the law of the pH effect varies. For example, when the pH value of NaH_2_PO_4_ increases, the conversion efficiency shows an upward trend. By contrast, the conversion efficiency declines as the pH of NaClO_4_ rises up. The results are probably influenced by the intrinsic properties of electrolytes. In total, the mechanism of the effects of electrolytes is still an open question remaining to be explored.

#### 2.2.3 Photoanodes

**Figure 6:**
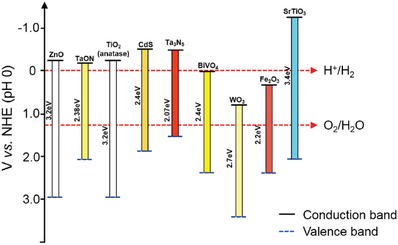
Schematic elucidation of bandgap positions of typical photoanodes.

Photoanodes are the decisive components for a PEC water oxidation system, which is in charge of absorbing solar energy, generating charge carriers, and then producing oxygen by oxidizing water. The oxygen evolving photoanode material should be an n‐type semiconductor. The band bending of it will generate an electric field and in turn drive holes to the surface of the materials. The stability under water oxidation conditions and the physicochemical properties like conductivity are also two main factors needed to be considered before selecting photoanodes in order to improve water splitting efficiency and reduce overpotential.^60^ Besides, the level of VB and CB as well as the width of bandgap are also very vital because the position of VB decides whether the water oxidation can proceed and the range of solar light absorption is influenced by the bandgap. Thus, a suitable bandgap (around 2.0–3.2 eV^61^) and an appropriate position of VB which is positive than the potential of O_2_ allow the oxygen production from water.^60^, ^62^ Common photoanodes for water oxidation include hematite, BiVO_4_, ZnO, TiO_2_, etc. **Figure** **6** shows the positions of VB and CB as well as the bandgap of representative photoanode materials.

In general, a photoanode consisting of a single semiconductor cannot satisfy the required performance of either photocurrent or conversion efficiency to meet commercial application due to their intrinsic limitations on performance and defects on properties, such as low solar light absorption, poor charge‐carrier transportation, and severe photocorrosion under illumination (**Table** **2**). Therefore, effective strategies such as surface modifications^63^ need to be applied to improve these properties of photoanodes. What is noteworthy is that designing functional photoanodes incorporated with cocatalysts to offset the drawbacks of single material is one of the states of art in PEC water splitting.^57^

**Table 2:** Advantages and disadvantages of common photoanode materials for PEC water oxidation

Photoanodes	Advantages	Disadvantages	Ref.
Fe_2_O_3_	Earth‐abundance; Nontoxicity; Good photochemical stability; Narrow bandgap (2.2 eV).	Low absorption coefficient; Short excited‐state lifetime (3–10 ps); Poor oxygen evolution kinetics; Short hole diffusion length (2–4 nm); Poor conductivity (≈10^−2^ cm^2^ V^−1^ s^−1^ at 20 °C).	4, 57, 61, 64, 65
WO_3_	Stable in acid conditions (pH < 4); Moderate hole‐diffusion length (≈150 nm); Highly tunable composition; Nonstoichiometric properties; Good electron transport properties.	Wide bandgap of 2.7 eV; Unstable when pH > 4; Corrosion induced by the peroxo‐species created during water oxidation.	4, 59, 61, 66
ZnO	Good stability; Environmentally friendly; Inexpensive; High carrier mobility.	Large bandgap of 3.2 eV; High recombination rate of electron–hole pairs.	4, 67
BiVO_4_	Suitable bandgap of 2.4 eV; Low onset potential for O_2_ evolution; Good stability.	Low IPCE at lower potentials; Poor photocurrent stability; Low intrinsic carrier mobility, due to short carrier diffusion length of ≈70 nm; Poor carrier separation efficiency; Slow hole kinetics of oxygen evolution.	61, 62, 68
TiO_2_	Highly stable over a wide range of pH values in aqueous environments upon illumination; Good catalytic properties; Appropriate valence band edges.	Large bandgap of 3.2 eV; Low conductivity.	4, 67, 69
TaON	Narrow bandgap 2.38 eV; Appropriate band positions; Environmentally benign.	Insufficient crystallization; Poor electrical conductivity; Self‐oxidative deactivation; Inefficient contacts among particles.	4, 61, 70
Ta_3_N_5_	Narrow bandgap 2.07 eV; Appropriate band positions; Environmentally benign.	Insufficient crystallization; Poor electrical conductivity; Self‐oxidative decomposition; The low carrier mobility Insufficient light absorption.	4, 30, 61
CdS	Moderate bandgap (2.4 eV).	Unstable in water upon illumination; Low charge separation and transfer efficiency.	4, 71

**Table** **3** shows the basic conditions and performances of photoanodes before and after the cocatalysts modification for water oxidation. It is evident that for most single semiconductor photoanodes, there is a significant gap between the practical photocurrent density and the theoretical ones. Whereas, after a photoanode is coupled with the cocatalyst, the current density multiplies and the aforementioned disparity shrinks in varying degrees. Hence, looking for suitable cocatalysts and coupling them with semiconductors are reasonable methods to increase the efficiency of PEC water oxidation.

**Table 3:** Summary of properties of common photoanode materials and PEC performance

Photoanodes	Bandgap [eV]	Bandgap wave length [nm]	Theoretical STHa) efficiency [%]^60^	Theoretical photocurrent density under 1 Sun irradiation 1.23 *V*_RHE_ b), 72 [mA cm^−2^]	Practical photocurrent density [mA cm^−2^]	Onset potential [mV, RHE]	Experimental conditions	Ref.
Fe_2_O_3_	2.20	564	12.9	10.6	0.20	0.84	1.23 *V*_RHE_, 0.5 m K‐Pi (pH = 7) under AM 1.5G (100 mW cm^−2^)	48
α‐Fe_2_O_3_/NiCoAl‐LDHc)					2.56	0.55		
BiVO_4_	2.40	517	9.1	7.5	1.20	0.70	1.23 *V*_RHE_, 0.2 m Na_2_SO_4_ (pH = 7), under AM 1.5G (100 mW cm^−2^)	73
β‐FeOOH/BiVO_4_					4.30	0.50		
TiO_2_ (Rutile)	3.00	413	2.2	1.8	0.79	0.50	1.23 *V*_RHE_, 0.5 m Na_2_SO_4_ (PH = 7), under AM 1.5G (100 mW cm^−2^)	74
TiO_2_/ZnFe‐LDH‐PEd) NAse)					1.51	0.35		
TiO_2_ (Anatase)	3.20	388	1.3	1.1	1.30	0.57	1.23 *V*_RHE_, 0.1 m Na_2_SO_4_, under AM 1.5G (100 mW cm^−2^)	75
TiO_2_ @CoNi‐LDHs NTAsf)					4.40	0.49		
WO_3_	2.70	459	4.8	4	0.56	0.62	1.23 *V*_RHE_, 0.1 m KNO_3_ (pH = 1.0), under AM 1.5G (100 mW cm^−2^)	76
WO_3_/Ir‐PO_3_H_2_					1.16	0.52		
CuWO_4_	2.30	540	10.8	9.0	0.58	0.30	0.8 *V*_RHE_, 1 m Na_2_SO_4_ (pH = 6.8), under AM 1.5G (100 mW cm^−2^)	77
CuWO_4_/CdS/FeOOH					2.05	0.20		
ZnO	3.20	388	1.3	1.1	0.04	0.76	1.23 *V*_RHE_, 0.5 m Na_2_SO_4_ (pH = 6.62), under AM 1.5G (100 mW cm^−2^)	67
NiO/ZnO					1.87	0.50		
TaON	2.38	521	9.9	7.7	0.80	0.65	0.6 *V*_Ag/AgCl_ g), 0.1 m Na_2_SO_4_ (pH = 6), under visible light (λ > 400 nm)	78
IrO_2_/TaON					3.80	−0.15		
Ta_3_N_5_	2.07	600	15.9	13.1	0.90	1.00	1.23 *V*_RHE_, 1 m NaOH (pH = 13.6), under AM 1.5G (100 mW cm^−2^)	79
Ni(OH)*_x_*/Ta_3_N_5_					5.40	0.68		

^a)^ STH: solar to hydrogen

^b)^ *V*_RHE_: Volt versus RHE

^c)^ LDH: layered double hydroxides

^d)^ PE: photo‐assisted electrodeposition (PED)

^e)^ NAs: nanoarrays

^f)^ NTAs: nanotubes

^g)^ *V*_Ag/AgCl_: Volt versus Ag/AgCl.

### 2.3 Important Parameters to Evaluate the Performance of PEC Water Oxidation

#### 2.3.1 Onset Potential and Photocurrent Density

Onset potential and photocurrent are two fundamental elements of a PEC process, which can help us rank the PEC performance of the obtained photoanodes. The lower the onset potential and higher photocurrent density, the better performance of photoanodes. When the proton energy absorbed by photoelectrodes is equal to or above the bandgap at these operating potentials (that is, *hv* ≥ *E*_g_), the OER at the electrode–electrolyte interface will be driven by a minority of hole carriers in n‐type electrodes while the HER will be initiated by a minority of electrons on the cathode surface. The potential at which OER/HER begins to happen under light is defined as the photocurrent onset potential (*E*_onset_). According to Ding et al.^17^ and Yang et al.,^80^ *E*_onset_ under irradiation will be influenced by two factors: open‐circuit photovoltage (*V*_ph_) and kinetic overpotential (η_k_), as Equation (4) shows. An increase of *V*_ph_ and a decrease of η_k_ could give rise to a cathodic shift of *E*_onset_ for a n‐type semiconductor

(4) $$E_{\mathrm{redox}}-\;E_{\mathrm{onset}}=V_{\mathrm{ph}}\;-\eta_{k}$$

where *E*_redox_ is the electrochemical potential of water oxidation and is irrelevant to the surface nature of the electrode.

As for photocurrent, it is the electric current passing through a PEC device as the response to light power and is directly proportional to the number of absorbed photons. If other parameters are held constant, the photocurrent depends linearly on the irradiation area. Thus, for the convenience of comparing the photoresponse performance of a solar cell with others, it is common to normalize the photocurrent via dividing it by the illuminated area (namely, photocurrent density, in the unit of mA cm^−2^). Furthermore, when comparing the capability of photoanodes, the photocurrent density at 1.23 *V*_RHE_ is an important benchmark point, since it is the theoretical potential where the oxygen evolution reaction can occur. Supposing only photons with energy larger than the bandgap of the semiconductor can be completely absorbed and all of the absorbed protons can be utilized for water splitting, then the photocurrent density will reach a maximum value, also known as the theoretical photocurrent density, which can be calculated by Equation (5)

(5) $$J_{\mathrm{phmax}}=\;e\int_{0}^{1240/E_{g}}I\left(\lambda \right)d\lambda$$

where *J*_phmax_ is the theoretical photocurrent density, *E*_g_ is the bandgap of the semiconductor, and *I*(λ) represents the photon flux at the wavelength of λ.

The theoretical photocurrent density is mainly determined by the bandgap of the semiconductor. Hence, it implicates the theoretical limit of a semiconductor to employ absorbed protons for water splitting. From Table 3, we can see that there is still a big gap between the practical photocurrent density and the theoretical one for most photoanode materials. Therefore, further research to optimize the performance of the photoelectrodes and approach the theoretical value of current density is quite indispensable.

**Figure 7:**
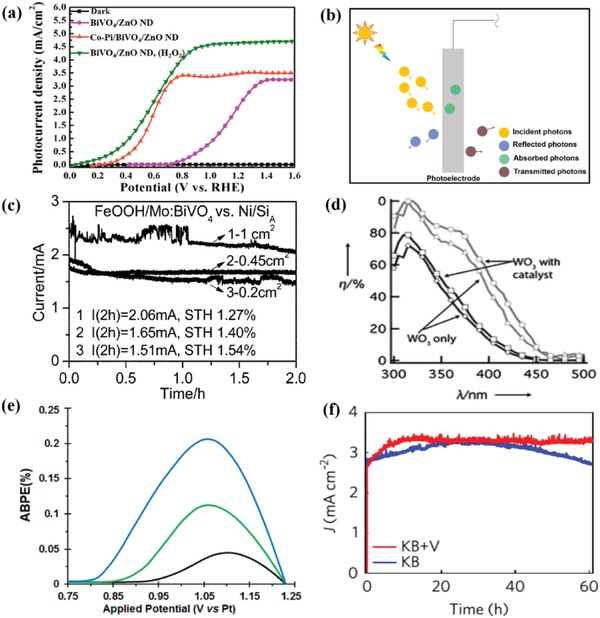
a) *J*–*V* plots of Co‐Pi/BiVO_4_/ZnO ND, BiVO_4_/ZnO ND, and BiVO_4_/ZnO ND with H_2_O_2_ in the electrolyte. Reproduced with permission.^68^ Copyright 2016, Elsevier. b) Schematic representation of photoelectrode after sun light irradiation. c) The STH efficiency (calculated from the average current in 2 h) grows from 1.27% to 1.51% when the area of cathode is decreased from 1 to 0.2 cm^2^. Reproduced with permission.^81^ Copyright 2014, Royal Society of Chemistry. d) The differences between the (APCE) (○ and △) and the IPCE (□ and ◇) of bare WO_3_ and WO_3_ incorporated with cocatalyst, respectively. Reproduced with permission.^82^ Copyright 2011, Wiley‐VCH. e) ABPE (%) of pure hematite on FTO glass (black), NF1‐P (green), and NF1‐NSP (blue) versus applied potential. Reproduced with permission.^83^ Copyright 2018, American Chemical Society. f) *J–t* plots of the BiVO_4_/FeOOH/NiOOH film in KB (blue) and KB+V (red) at 0.6 *V*_RHE_ (AM 1.5G, 100 mW cm^−2^; scanning rate:10 mV s^−1^). Reproduced with permission.^84^ Copyright 2017, Springer Nature.

Both the onset potential and photocurrent density can be demonstrated via liner sweep voltammetry (LSV), which is a voltammetric technique where electric current between working and counter electrode is measured while linearly sweeping the potential of the working electrode in the positive direction. It is also referred to as *J*–*V* measurements (photocurrent density vs voltage). In most cases, the data will be recorded in the dark conditions and under simulated solar light illumination (AM 1.5G, 100 mW cm^−2^) at a certain scan rate. As illustrated in **Figure** **7**a, the LSV plot under dark scarcely exhibits any current.^68^ Whereas, all the samples show photoresponse to the radiance after irradiation. Specifically, after the introduction of Co–Pi onto the BiVO_4_/ZnO ND photoanode, it emerges as much higher PEC performance than BiVO_4_/ZnO nanodendrite (ND). The photocurrent density at 1.23 *V*_RHE_ rises from 2.45 to 3.5 mA cm^−2^, the saturated photocurrent density rises from 3.25 (at 1.35 V) to 3.5 mA cm^−2^ (at 0.8 V), and the onset potential negatively shifts from 0.7 *V*_RHE_ to 0.3 *V*_RHE_, indicating that the performance of BiVO_4_/ZnO ND can be optimized by the cocatalyst Co–Pi significantly.

#### 2.3.2 PEC Efficiency

Multiple methods can be used to measure PEC efficiency and help us gain more insight into material performance. Usually these methods can be divided into two main categories: 1) Benchmark efficiency (suitable for mainstream reporting of stand‐alone water splitting capability) like solar‐to‐hydrogen (STH) conversion efficiency and 2) diagnostic efficiencies (to characterize and understand materials system or interface performance), such as incident photon‐to‐current efficiency (IPCE), absorbed photon‐to‐current efficiency (APCE), and applied bias photon‐to‐current efficiency (ABPE). IPCE covers the conversion efficiency of absorbed light and the degree of light absorption while APCE only considered the effects of absorbed light. As shown in Figure 7b, after illumination, only part of the incident photons can be absorbed in photoelectrode. Thus, by definition, the value of IPCE is always lower than that of APCE. Meanwhile, ABPE was measured under bias, so the value is closer to real experiment conditions. Despite that these methods are all used to evaluate the efficiency of a PEC device, they are calculated by different formulas and each have dissimilar emphasis. Hence, they are all unique in PEC research.^36^, ^56^

*STH Efficiency*: STH efficiency is the most essential and practical parameter to characterize the water splitting ability of a PEC cell among all the efficiency metrics. It describes the water splitting efficiency of a PEC cell illuminated by broadband sunlight under zero bias conditions and it is the only device efficiency that can be commonly used to act as a reliable reference to rank a PEC device against others.^85^ A STH efficiency higher than 10% can be used for practical application.^86^ Table 3 lists the STH efficiency of some typical photoanodes, and it shows the efficiency of photoanodes that can be achieved up to now is still far from being qualified for commercial use.

The standard definition for STH efficiency under 1 sun condition can be expressed by Equation (6). The numerator is the output energy of H_2_ based on the rate of H_2_ evolution directly measured by analytical methods, which equals to the rate of hydrogen production (mmol s^−1^) multiplied by the change in Gibbs energy per mole of H_2_ (that is, Δ*G*_0_ = 237 kJ mol^−1^ at 25 °C). And the denominator is the incident solar energy input from sunlight which equals to *P*_total_ (solar energy flux, mW cm^−2^) multiplied by the surface area (cm^2^) of the irradiated electrode.^36^, ^56^, ^87^ Figure 7c shows the STH efficiency of FeOOH/Mo:BiVO_4_ versus Ni/Si_A_ as an example. As can be seen, the STH efficiency climbs up from 1.27% to 1.54%, while the Si_A_ area declines from 1 to 0.2 cm^2^. As the output power is decided by the thermodynamic water splitting potential (1.23 eV at 25 °C) based on Δ*G*_0_, the short‐circuit photocurrent density (*j*_SC_), and the Faradaic efficiency (η_F_) for hydrogen evolution, the STH efficiency can also be expressed by Equation (7) as a substitute^36^, ^56^

(6) $$\mathrm{STH}\;={\left[\frac{H_{2}\left(\mathrm{mmol}\;s^{-1}\right)\times \;237\;000\left(J\;{\mathrm{mol}}^{-1}\right)}{P_{\mathrm{total}}\left(\mathrm{mW}\;{\mathrm{cm}}^{-2}\right)\times \;\mathrm{Area}\left({\mathrm{cm}}^{2}\right)}\right]}_{\mathrm{AM}\;1.5G}$$

(7) $$\mathrm{STH}\;={\left[\frac{\left|j_{\mathrm{SC}}\left(\mathrm{mA}\;{\mathrm{cm}}^{-2}\right)\right|\times \;1.23\left(\mathrm{eV}\right)\times \eta_{F}}{P_{\mathrm{total}}\left(\mathrm{mW}\;{\mathrm{cm}}^{-2}\right)}\right]}_{\mathrm{AM}\;1.5G}$$

It needs to be emphasized that Equations (6) and (7) can be used only if it is confirmed that the water splitting reaction proceeds without any sacrificial electron donor or acceptor. Once they are utilized, water will not be split, and the above equations for STH efficiency become meaningless.^36^, ^56^

*IPCE*: The IPCE, which is synonymous with the external quantum efficiency (EQE), describes the photocurrent collected per incident photon flux due to the electrons moving through an external circuit as a function of irradiation wavelength. It is independent of the light sources used in the experiment and is one of the most crucial diagnostic tools to understand the inherent performance of a PEC photoanode.^36^, ^56^

The IPCE can be calculated using Equation (8) at a given bias voltage

(8) $$\begin{array}{c} \mathrm{IPCE}\;\left(\%\right)=\;\mathrm{EQE}\;\left(\%\right)=\frac{\mathrm{electrons}\left({\mathrm{cm}}^{2}\;s^{-1}\right)}{\mathrm{photons}\left({\mathrm{cm}}^{2}\;s^{-1}\right)}\;\\ \times \;100\;=\frac{1240\left(V\;\times \;\mathrm{nm}\right)\;\times \left|j_{\mathrm{ph}}\left(\mathrm{mA}\;{\mathrm{cm}}^{-2}\right)\right|}{\lambda \left(\mathrm{nm}\right)\;\times P_{\mathrm{mono}}\left(\mathrm{mW}\;{\mathrm{cm}}^{-2}\right)}\;\times \;100 \end{array}$$

where 1240 (V × nm) equals to *h* (Planck's constant, 6.63 × 10^−34^ J s) multiplied by *c* (the speed of light, 3 × 10^8^ m s^−1^) and *e* (the charge of an electron, 1.6 × 10^−19^ J eV^−1^); *j*_ph_ is the measured photocurrent density (mA cm^−2^); λ represents the wavelength of incident light (nm); *P*_mono_ is the calibrated intensity of the incident light (mW cm^−2^).^36^, ^61^

IPCE plays an extremely important role in evaluating PEC water splitting properties of photoanodes, as it calculates the efficiency in the form of “electrons out per photons in” and considers the influences of spectral change of incident photons. In the process of water splitting, IPCE can describe the upper limit of efficiency to produce hydrogen or oxygen from water on condition that all electrons and holes are used. However, when possible photocorrosion or side reactions occurs, the IPCE values may be higher than the virtual ones.^36^

*APCE*: APCE is identical to internal quantum efficiency (IQE). It measures the efficiency based on the absorbed incident photons, assisting researchers to understand the inherent properties of a material. When we test the performance of thin films, APCE is particularly important, as it helps to find the balance between maximal path length for photon absorption and minimal effective *e^−^*/*h^+^* transport distance within the material. The calculation of APCE can be derived as Equation (9).^36^ The differences between IPCE and APCE with or without cocatalyst can be seen in Figure 7d as an example. By coupling cocatalyst, the absorption efficiency is promoted when the charge collection capability of photoelectrode is ensured simultaneously

(9) $$\mathrm{APCE}\;\left(\%\right)=\;\mathrm{IQE}\;\left(\%\right)=\frac{1240\left(V\;\times \;\mathrm{nm}\right)\;\times \left|j_{\mathrm{ph}}\left(\mathrm{mA}\;{\mathrm{cm}}^{-2}\right)\right|}{\lambda \left(\mathrm{nm}\right)\;\times P_{\mathrm{mono}}\left(\mathrm{mW}\;{\mathrm{cm}}^{-2}\right)\;\times \left(1-10^{-A}\right)}\;\times \;100$$

where 1240 (V × nm) equals to *h* (Planck's constant) multiplied by *c* (the speed of light) and *e* (the charge of a electron, 1.6 × 10^−19^ J eV^−1^); *j*_ph_ is the measured photocurrent density (mA cm^−2^); λ represents the wavelength of incident light (nm); *P*_mono_ is the intensity of the incident light (mW cm^−2^); *A* represents the absorbance of a sample, which can be estimated from Beer's Law.

*ABPE*: ABPE is of particular interest because STH cannot reflect a true PEC water splitting process when a bias is applied.^36^ It can further elucidate the diagnostic performance of the employed photoelectrodes and also show the net production of current through the extraction of electrons from the electrode by applying a suitable bias voltage. The following formula can be employed to calculate the ABPE in a two‐electrode configuration (working electrode and counter electrode)

(10) $$\mathrm{ABPE}\left(\%\right)\;=\frac{\left|J_{\mathrm{ph}}\left(\mathrm{mA}\;{\mathrm{cm}}^{-2}\right)\right|\times \;\left(1.23-\left|V_{b}\right|\right)\left(V\right)}{P_{\mathrm{total}}\left(\mathrm{mV}\;{\mathrm{cm}}^{-2}\right)}\;\times \;100$$

where *J*_ph_ is the obtained photocurrent density under an applied bias *V*_b_ (mA cm^−2^). *V*_b_ equals applied bias (V); *P*_total_ is the illumination intensity of the light source (mW cm^−2^) and *V* is the open circuit potential recorded from the *J*–*V* curve under illumination.^88^

Figure 7e depicts the ABPE performance of the optimized NiFe_2_O_4_/Fe_2_O_3_ thin films deposited on planar (NF1‐P) and 3D‐nanostructured substrates (NF1‐NSP) relative to pure Fe_2_O_3_ deposited on FTO glass under solar irradiation (100 mW cm^−2^). The peak ABPE value of NF1‐NSP was 0.206% located at 1.06 V, 1.87 times higher than NF1‐P and 4.7 times higher than pure Fe_2_O_3_, which means NF1‐NSP has better performance than the pure Fe_2_O_3_.

#### 2.3.3 Photostability

Photostability is defined as the capability of photocorrosion inhibition. Photostability test is also fundamentally significant to evaluate the durability of a photoanode material. In general, the photostability of a photoanode can be measured by *J–t* curves (photocurrent density vs time) under illumination with a certain bias between the working electrode and the counter electrode. A bias that could lead to photocurrent densities corresponding to anticipated PEC operations (typically 1–10 mA cm^−2^) will be a suitable bias for testing. For example, Figure 7f describes the *J*–*t* measurement of BiVO_4_/FeOOH/NiOOH photoelectrode in potassium borate buffer (KB) (blue) and KB + V (V_2_O_5_) (red) solution. The photocurrent density of BiVO_4_/FeOOH/NiOOH film in KB solution shows an upward trend for about 20 h and then decreases gradually after about 40 h, indicating that the photoanode is not completely photostable over a long time. Correspondingly, the photocurrent density of BiVO_4_/FeOOH/NiOOH film in KB+V solution gradually rises to 3.2 ± 0.2 mA cm^−2^ during the first 10 h and is maintained for 50 h, suggesting that photocorrosion is suppressed due to the addition of V_2_O_5_ to the electrolyte.

#### 2.3.4 Conductivity

The conductivity of the PEC system determines the charge transfer efficiency in the electric circuit. Usually, it can be measured by electrochemical impedance spectroscopy (EIS), which is one of the universal and powerful modulation techniques and is helpful for further understanding the complex reaction at the photoelectrode surface. It allows sample characterization in electrolyte under bias potential, which is one of the major advantages of EIS compared with other spectroscopic techniques.

**Figure 8:**
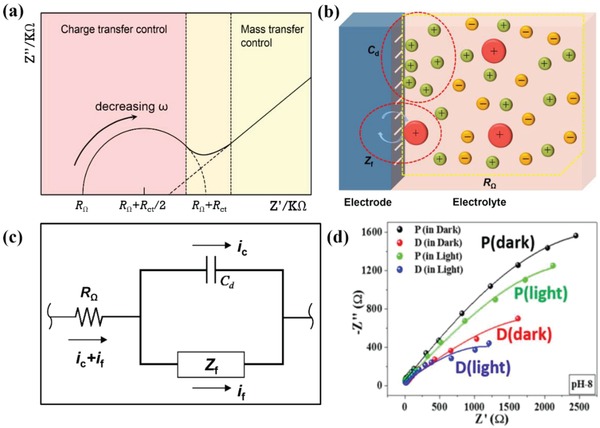
a) A typical Nyquist plot of a photoelectrode in the photo‐electrochemical process. ω is the angular frequency. b) The constitutes of total impedance. c) A simplified equivalent resistor‐capacitor (RC) circuit model. d) Nyquist plots of the sample P and sample D at pH 8. Reproduced with permission.^88^ Copyright 2017, Elsevier.

EIS experiments are composed of applying a small‐amplitude sinusoidal signal to the photoanodes at a certain bias and measuring the response of the system to the perturbation. During an EIS experiment, the current density of the electrode is recorded while applying an alternating current signal with different frequencies (usually 0.1 Hz < *f* < 10^6^ Hz) to the system.^89^ By EIS characterization, Nyquist plot (real vs imaginary impedance) can be obtained. In the spectrum, the *X*‐axis is the real impedance (*Z′*, in the unit of Ohms), while the *Y*‐axis represents the imaginary impedance (*Z″*, in the unit of Ohms).^90^ Theoretically, a typical Nyquist plot can be divided into two parts: high frequency region and low frequency region, as shown in **Figure** **8**a.^90^ High frequency region can be observed close to the original point of the plot. In this region, the photo‐electrochemical process is controlled by the rate of charge transfer and the curve exhibits the shape of a semicircle which hints the existence of an interface. In low frequency region (large impedance, far from the origin), the process is determined by the rate of mass transfer. When the applied frequency approaches zero, the *Z′* equals to *Z″*, and the curve turns into a straight line with a slop of 1.^30^, ^89^, ^90^ Sometimes, there are more than one semicircles on the Nyquist plot. Gomes and Vanmaekelbergh^91^ ascribe these semicircles to a multiple‐step electron‐ or charge‐transfer process mediated via surface states or reaction intermediates. In the actual EIS experiments, the curves at the Nyquist plot tend to display a shape of distorted semicircle or a straight line whose slop is not equal to 1, because the shape of the curves could be affected by the intrinsic or electrochemical properties of electrodes, electrolytes, and the conditions of the experiments.^92^

For the spectrum, the charge‐transfer resistance (*R*_ct_) is in direct proportion to the chord length of the arc. The larger chord length, the larger *R*_ct_ will be, which also indicates its poor charge‐transfer ability or conductivity for water splitting.^30^ Furthermore, the capacitance can be determined by fitting the frequency responses with an equivalent resistor‐capacitor (RC) circuit model.^89^ As shown in Figure 8b, at the interface between electrode and electrolyte in a PEC water splitting process, there are inner resistances of electrolyte and photoelectrodes (*R*_Ω_), double layer capacitance (*C*_d_, derived from nonreactive ions in the electrolyte which only change the charge distribution), and Faradaic impedance (*Z*_f_, derived from the active ions in the electrolyte, which leads the charge transfer), so an equivalent circuit model can be established based on them to indicate the conductivity of the entire system.^90^ A simplified model is shown in Figure 8c.

Moreover, the light intensity applied to the PEC system will largely influence the impedance spectra. The Nyquist plots of the sample P (pristine Fe_2_O_3_) and sample D (Co‐Ac/Ti‐Fe_2_O_3_) at pH 8 are shown in Figure 8d. The arc of them shows the similar trends but different diameters. It indicates that the internal resistance which determines the rate of charge transfer in the dark is significantly larger than that under light conditions. This resistance is derived from the charge equilibrium, which leads the electrons to accumulate in the electrodes.^93^, ^94^ Whereas, when under illumination, the electrons stored in the conduction band will transfer to the external circuit because of the photoexcitation and then lower the impedance of the system. Furthermore, it is noteworthy that for the same impedance spectrum, more than one equivalent RC circuit models can be used to fit the frequency responses. Hence, it is a subjective approach to speculate a PEC process by an equivalent circuit model.

### 2.4 Cocatalysts

The water oxidation half reaction (Equation (2)) is a fundamental element of water splitting, but it is more complex than water reduction. Therefore, this half reaction is regarded as the most challenging step in the artificial PEC water splitting. To significantly improve the activity of photoanodes, coupling the suitable cocatalysts has been regarded as a very potential and effective approach.

**Figure 9:**
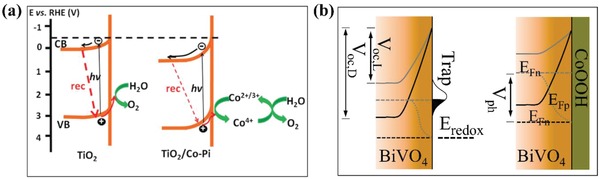
a) Schematic diagram of band bending with and without Co–Pi on TiO_2_ for PEC water oxidation. Reproduced with permission.^102^ Copyright 2015, Royal Society of Chemistry. b) Photovoltaic response on BiVO_4_ (left) and BiVO_4_/CoOOH (right) under dark (black lines) and light (grey lines). After coating CoOOH, the surface trap states of BiVO_4_ is eliminated. *V*_oc,L_ is open‐circuit voltage in the dark; *V*_oc,D_ is open‐circuit voltage under irradiation; *E*_redox_ is the potential of water splitting redox reaction. *V*_ph_ is the relevant photovoltage; *E*_Fp_ and *E*_Fn_ are quasi‐Fermi level of holes and electrons respectively. Reproduced with permission.^103^ Copyright 2018, American Chemical Society.

Cocatalyst is a kind of special catalyst which does not have optical absorption property and cannot improve the light harvesting directly but is highly conductive and plays an indirect role in improving the efficiency of PEC water oxidation.^95^ It can be an amorphous structure with enriched reaction sites or a continuous layer with certain crystallinity. Coupling proper cocatalyst with photoanode is an effective method to enhance the PEC water splitting performance. However, the functions of cocatalysts still remain disputed:

1) Cocatalysts are conducive to promoting the separation of electron–hole pairs due to the ability to change the distribution of photogenerated carriers.^95^ After engineering a water oxidation cocatalyst on the photoanode, an intimate junction at the interface will be formed. Then, an enhanced upward band bending occurs due to the difference between the cocatalyst and electrode (**Figure** **9**a), leading electrons to be depleted in the space charge region and excessive holes to occur on the surface of the semiconductor. Then due to the hole capture sites on the cocatalyst, the photogenerated holes of semiconductor will transfer to the cocatalyst through the junction, which provides a shortcut for their transfer for oxidizing water rather than accumulating on the surface. This process could reduce the surface hole concentration of the semiconductor and relieving the pinned quasi‐Fermi level to a certain degree which in turn reduces surface recombination.^96^, ^97^, ^98^
2) Cocatalysts could increase the stability of photocatalysts physically and suppress the photocorrosion. They can accelerate the reaction process and consume the left photoinduced carriers, especially the holes so as to avoid photocorrosion. For instance, (oxy)sulfides and (oxy)nitrides are easily oxidized by photogenerated holes, resulting in their self‐decomposition. Whereas, loading cocatalysts thereon can suppress the decomposition by extracting the photogenerated holes for O_2_ evolution, thereby enhancing the robustness of semiconductors.^97^ Furthermore, cocatalysts could also remove the surface trapping states of photoanodes (Figure 9b), which could cause substantial recombination on the surface and pin the Fermi level, and then ensure the stability of the system.^99^, ^100^
3) In a PEC water oxidation process, cocatalysts possess numerous reaction sites, which can lower the overpotential of O_2_ evolution reactions on the surface of semiconductors, give rise to a cathodic shift of onset potential, enhance the kinetics of water oxidation, and then largely improve the PEC water oxidation performance.^18^, ^97^, ^101^

In total, a multitude of researches have proved that coupling cocatalysts is an effective solution to enhance PEC water oxidation performance and the feasible mechanisms are also explained from different perspectives. Whereas, it is also undeniable that some of the explanations about how cocatalysts work still remain vague, thus it is a must to conduct further research and come up with more clear clarifications to assist us in having insight into the virtual mechanism.

## 3 Cocatalysts for Photoanodes: Construction and Regulation

### 3.1 Metal Phosphates/Phosphides

#### 3.1.1 Metal Phosphates

Metal phosphates, especially cobalt phosphates (Co–Pi), are the pioneer cocatalyst candidates applied in PEC‐OER process, as they are earth‐abundant, amorphous, ion permeable, easy to form on diverse surfaces of varying semiconductor materials, and self‐healing when in the presence of phosphate buffer solution. Besides, they can also be favorable for catalyzing water splitting over a wide pH range of 1–14,^102^ making them viable alternatives to noble‐metal‐based cocatalysts.

In 2008, the formation of amorphous Co–Pi was first reported by Kanan and Nocera.^104^ After that, Steinmiller and Choi^105^ and Zhong et al.^106^ also reported the excellent capability of Co–Pi in solar water splitting. Since then, this catalyst has gained numerous interests and become one of the research hotspots. It is reported that Co–Pi can change the reaction pathway to reduce overpotential and promote surface charge transfer kinetics.^107^ Co^2+/3+^ of Co–Pi can be oxidized to a higher valence state of Co^4+^ under the effect of current or holes, and then Co^4+^ can be reverted back to Co^2+/3+^ rapidly after it oxidized water molecules or sacrificial agents,^50^ which is recognized as an entire catalytic reaction cycle. Meanwhile, as the change in valence state of Co ion is invertible, the accumulation of photoinduced holes at the interface of electrode and electrolyte can be relieved timely and effectively and then an improved PEC performance can be achieved.^102^

Recently, in‐depth study about Co–Pi for PEC water oxidation has been carried out. Some of the researchers focus on the interaction of Co–Pi and traditional photoanodes, such as α‐Fe_2_O_3_, BiVO_4_, TiO_2_, etc.^96^, ^102^, ^108^, ^109^, ^110^, ^111^, ^112^ Some further clarified the mechanism of the Co–Pi applied on semiconductors.^96^, ^109^ For example, Carroll and Gamelin^96^ reported that water‐oxidation kinetics of α‐Fe_2_O_3_ actually gets slow after the deposition of Co–Pi, but electron–hole recombination slows even more, resulting in a net enhancement of water‐oxidation quantum efficiency, which is opposite to previous expectations. Moreover, the methods have also been investigated for optimizing the effects of Co–Pi in terms of experiment conditions such as quantity (i.e., thickness),^102^, ^108^ deposition time,^112^ pH values of the solution,^102^ applied bias potential,^110^ or morphology of the semiconductors.^111^ For instance, Co–Pi was coated onto BiVO_4_ photoanode via a PED (photoelectron deposition) technique by Hernández et al.^110^ They varied the bias and time for PED to maximize the Co–Pi activity and found that a combination of 1.32 *V*_RHE_ and 60 s for PED could contribute to an optimum amount of Co–Pi on BiVO_4_ and an improved photocurrent of up to 3 mA cm^−2^ at 1.23 *V*_RHE_ at pH 7.

**Figure 10:**
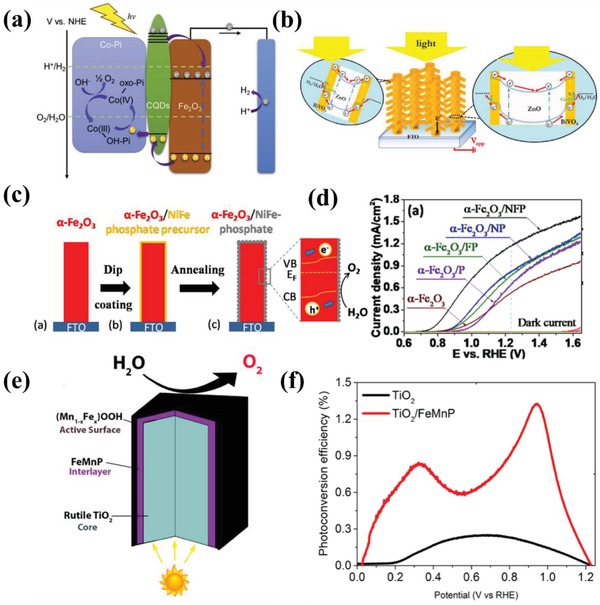
a) Charge separation and transfer mechanism in the C–Co–Pi/Fe_2_O_3_ photoanode. Reproduced with permission.^114^ Copyright 2018, Elsevier. b) Schematic of Co–Pi/BiVO_4_/ZnO ND photoanode. Insets are the band diagrams of trunk and branch of Co–Pi/BiVO_4_/ZnO ND. Reproduced with permission.^68^ Copyright 2016, Elsevier. c) Schematic diagram of the fabrication process and mechanism of α‐Fe_2_O_3_/NFP nanoarrays. d) *J−V* curves of pure α‐Fe_2_O_3_ and α‐Fe_2_O_3_ loaded with different catalysts 0.1 m KOH solution under AM 1.5G (100 mW cm^−2^). Reproduced with permission.^117^ Copyright 2017, American Chemical Society. e) The structure of TiO_2_/FeMnP core/shell photoanode for PEC water oxidation. f) The photoconversion efficiency of pure TiO_2_ and TiO_2_/FeMnP. Reproduced with permission.^118^ Copyright 2017, American Chemical Society.

In order to further optimize the PEC performance of catalysts, some ternary composites containing Co–Pi like Fe_2_O_3_/CdS/Co–Pi^50^ and BiVO_4_/ZnO/Co–Pi are also proposed.^113^, ^114^, ^115^, ^116^ These ternary catalysts exhibit much more prominent water oxidation capability due to the intimate contact of three composites and the synergistic effects among them. In most cases, the intermediate material between the photoanode and the Co–Pi works as a charge bridge between them^114^, ^115^, ^116^ (such as what **Figure** **10**a shows) or an electron trap^68^ (like Figure 10b) to accelerate the charge separation. For instance, Yang and Wu^68^ reported a Co–Pi/BiVO_4_/ZnO ternary system for water oxidation. As shown in Figure 10b, the photogenerated holes drift to central portions of ZnO trunk and branches and then be depleted while the holes can be collected by Co–Pi on the surface of BiVO_4_, which significantly promotes the charge separation efficiency and the PEC performance (3.5 mA cm^−2^ at 1.23 *V*_RHE_ under 1 sun light irradiation).

Except for Co–Pi, other metal phosphates,^117^, ^119^, ^120^, ^121^, ^122^ such as Ni–Pi^117^ and Fe–Pi^121^ are also observed to be capable of facilitating PEC water oxidation. For instance, Gao and co‐workers^119^ employed a deposit‐PEC method to incorporate Ni–Pi with Ti doped α‐Fe_2_O_3_, which showed enhanced PEC water oxidation activity. It also reveals that Ni–Pi could restrain surface states which attribute to Fe^3+^/Fe^2+^ redox couples in oxygen deficient regions and will trap holes or electrons on the surface, causing serious recombination,^121^ while Co–Pi could not in this system. Liu et al.^117^ described the synthesis of α‐Fe_2_O_3_ nanoarrays photoanode with ultrasmall NiFe‐phosphate (NFP) nanoparticles via a facile dip‐coating and annealing method (Figure 10c). The NFP nanoparticles have the combined effects of Ni–Pi and Fe–Pi and show higher catalytic activity due to the synergistic effect on promoting the multiphoton‐coupled electron transfer process for the PEC water oxidation (Figure 10d).

#### 3.1.2 Metal Phosphides

Metal phosphides typically have a metallic nature, and the introduction of metal phosphide to the surface of semiconductors could promote the interfacial charge transfer and transportation to the surface. Previously, metal phosphides have been extensively investigated as outstanding OER electrocatalysts, while their application in the PEC‐OER process has rarely been reported. Recently, some metal phosphides have been found to work as PEC‐OER cocatalysts. For example, Wang et al.^123^ reported that GaN:ZnO/NiCoFeP showed high efficiency for PEC O_2_ evolution. With NiCoFeP, the surface charge injection is greatly promoted and the photocurrent density at 1.23 *V*_RHE_ reached up to 3.9 mA cm^−2^.

In spite of the high activity, the demanding preparation conditions in terms of high temperature and the strong reductive ability are still the biggest issues that hinder their extensive application. Confronted with it, researchers have made some efforts to tackle this bottom‐neck. Among them, an ideal technique named metal–organic chemical vapor deposition was designed to grow metal phosphide onto semiconductors directly under mild conditions. With this method, Schipper et al.^118^ successfully synthesized an efficient TiO_2_/FeMnP core/shell photoanode for PEC water oxidation, which reached the theoretical photocurrent density for rutile TiO_2_ of 1.8 mA cm^−2^ at 1.23 *V*_RHE_ under 1 sun irradiation (Figure 10d). The structural schematic diagram and photoconversion efficiency can be seen in Figure 10e,f, respectively. Enlightened from the bifunctional effects of metal phosphide in the field of electrochemistry, some researchers also explored their dual function in PEC water splitting. Kim et al.^124^ reported a CoP‐coupled Mo:BiVO_4_ photoanode connected with CoP‐coupled Ni‐foam synthesized by drop casting, exhibiting excellent PEC‐OER and PEC‐HER activities. And the CoP loading brings ≈90% injection of holes arriving on the surface, which avoided the serious recombination and largely boosted the kinetics of reaction.

### 3.2 Metal Oxides

Metal oxides such as IrO*_x_*, RuO*_x_*, NiO*_x_*, CoO*_x_*, and other hybrid metal oxides are promising materials as cocatalysts for the PEC applications, especially for the water oxidation, due to their easy‐established preparation, high stability, and activity facilitating water oxidation.

#### 3.2.1 Noble Metal Oxides

Noble metal oxides such as RuO_2_ or IrO_2_ are regarded as one of the most active cocatalysts, and exhibit excellent metallic electronic conductivity.^125^ Therefore, they are recognized as very promising candidates for PEC water splitting theoretically. Majumder and Khan^126^ revealed an obvious onset potential shift of ≈120 mV using α‐Fe_2_O_3_ photoanode coupled with RuO_2_ for PEC water oxidation. Tilley et al.^127^ reported the synthesis of IrO_2_‐loaded α‐Fe_2_O_3_ photoanode via an electrochemical deposition strategy. It is the first time that the photocurrent density of α‐Fe_2_O_3_ photoanode at 1.23 *V*_RHE_ could surpass 3 mA cm^−2^. Apart from above, Ag‐based catalysts (AgCat) are also very potential for promoting water oxidation due to their high activity. In 2017, Sordello et al.^128^ first reported that when coupling AgCat with TiO_2_ and hematite separately, the existence of AgCat can help produce much higher photocurrent density and O_2_ production compared with bare semiconductors or Co–Pi modified ones.

However, these noble‐metal based materials are not perfect cocatalysts due to some defects. For example, RuO_2_ exhibits a weak ability to resist corrosion under alkaline conditions and IrO*_x_* possesses relatively poor conductivity. Furthermore, the most nonnegligible issue is their high cost, extremely limiting their large‐scale application. Thus, it is highly necessary to find highly active cocatalysts with good stability but low cost as alternatives of these precious metal‐based cocatalysts, and the earth‐abundant transition‐metal oxides should be one of the best choices.

#### 3.2.2 Earth‐Abundant Transition‐Metal Oxides

In the recent years, earth‐abundant transition‐metal oxides gradually emerge with their advantages and become good alternatives due to their low cost and outstanding ability to resist chemical‐corrosion or photocorrosion in certain electrolytes. And among these materials, cobalt‐ and nickel‐based oxides are recognized as the promising PEC‐OER cocatalysts.

*CoO_x_*: CoO*_x_* is very popular as an excellent cocatalyst. It has been confirmed that CoO*_x_* is capable of reducing reaction activation energy and forming internal built‐in electric field which can boost the electrons transfer number at the same time. In addition, other merits of utilizing CoO*_x_* as the cocatalysts include the selectivity improvement of water oxidation, the large Faradaic efficiency enhancement of the reaction process, and the higher stability.^129^ Up to now, CoO*_x_* has been widely applied in PEC OER water oxidation.^130^, ^131^, ^132^, ^133^, ^134^, ^135^, ^136^, ^137^, ^138^, ^139^, ^140^, ^141^ For example, Ti/Fe_2_O_3_/CoO*_x_* was successfully designed for PEC water oxidation^137^ and the photocurrent could achieve an obvious enhancement compared with Ti/Fe_2_O_3_, resulting from the construction of a metallic charge transfer channel between the photoanode and cocatalyst, which could accelerate the flow speed of holes. Apart from inorganic materials, CoO*_x_* was also reported to be anchored onto organic molecules for oxygen evolution. CoO*_x_* incorporated perylene diimide was first prepared by Kirner and Finke for PEC water oxidation,^133^, ^140^ which made the hypothesis of using organic molecules in water oxidation come true.

**Figure 11:**
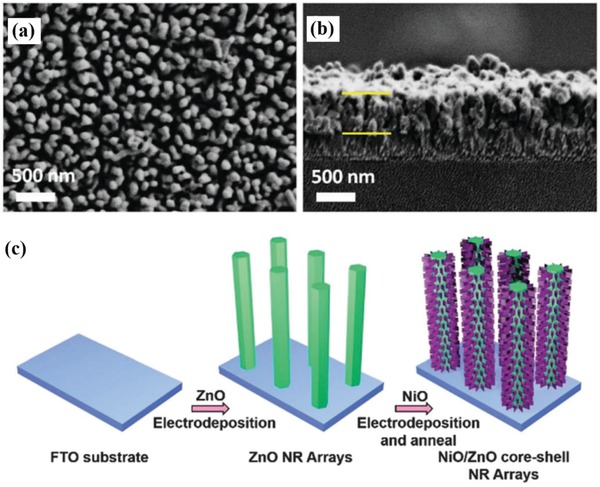
a) Top‐view and b) side‐view SEM images of Fe_2_O_3_ with 25 cycles of ALD CoO*_x_* coating. Reproduced with permission.^139^ Copyright 2017, Royal Society of Chemistry. c) Schematic procedure to synthesize NiO/ZnO core–shell NR arrays on the FTO glass. Reproduced with permission.^67^ Copyright 2016, Royal Society of Chemistry.

Generally, the method to deposit cobalt oxides onto photoanodes was tuned, which realized the modulation of structure and the improvement of PEC performance. Xi et al.^136^ revealed an in situ incorporation strategy to couple Co_3_O_4_ with Fe_2_O_3_ and the composite exhibited preferable capabilities compared to those prepared by ex situ procedures due to higher surface roughness, larger interfacial area, and smaller particle size. Meanwhile, an atomic layer deposition (ALD) method was also applied to couple hematite with an ultrathin CoO*_x_* layer at low deposition temperature, the morphology of which after 25 cycles of ALD can be seen in **Figure** **11**a,b.^139^ The assembled composite inhibits the recombination and achieves a high IPCE increment of 66% at 1.23 *V*_RHE_. In addition, Zhan et al.^130^ reported a WO_3_/CoO*_x_* photoanode for water oxidation with a dipping annealing strategy. The result shows the outstanding catalytic performance of WO_3_/CoO*_x_* photoanode, with IPCE value reaching 49.1% from 24.9%. Another common technique is impregnation.^132^, ^135^ CoO*_x_* fine particles was deposited on Ta_3_N_5_ electrode by Niishiro et al.^135^ via this way, which conspicuously improved the durability and reaction efficiency of CoO*_x_*/SnNb_2_O_6_.

A suitable deposition method can modulate CoO*_x_* with improved morphologies and better performance. Besides, other strategies can also boost the capability of cobalt oxides. For example, metal–organic framework (MOFs) technology is used to enhance the activity of CoO*_x_*. MOFs‐derived porous Co_3_O_4_ sheets were employed to modify TiO_2._ 141 The prepared photoanode shows fair stability and achieves a photocurrent density of 2.71 mA cm^−2^ at 1.23 *V*_RHE_ in alkaline electrolyte on account of the large specific surface area and enriched pore structure originated from MOFs.

*NiO_x_*: Nickel oxide (NiO*_x_*) is viewed as one of the best OER cocatalysts as it is earth‐abundant, cost‐efficient, stable even at high pH, and optically transparent under visible light, which enable the deposited photoanodes possess high durability and excellent water oxidation performance.^134^ In 2012, it is found by Sun et al.^142^ that when incorporated with n‐type silicon (n‐Si), the NiO*_x_* thin film can be utilized as the cocatalyst to resist photocorrosion and achieve high oxygen evolution efficiency under neutral pH condition, which brought about some possibilities for nickel oxide to be applied in PEC water oxidation. Subsequently, Sun et al. further investigated the properties of sputtered NiO*_x_* films,^143^, ^144^, ^145^ and found the NiO*_x_* layer is a conductive and chemically stable coating that can stabilize the photoanode and boost the PEC water oxidation efficiency simultaneously. All their work gives a large impetus to the further research of NiO*_x_* serving as a cocatalyst.

Recently, an amorphous NiO cocatalyst was induced onto ZnO photoanode by a method of two step electrondepositions and annealing,^67^ as shown in Figure 11c. This core/shell hybrid broke the efficiency limit of pristine ZnO and achieved a remarkable 260 mV negative shift of onset potential for photo‐oxidation of water. Another common method named metal–organic chemical vapor deposition strategy was applied to deposit NiO onto GaN. The band alignment between them offered efficient carrier separation and fast hole transport, making its PEC performance significantly outperform that of pure GaN.^146^ Besides, an n‐silicon photoanode coated by a NiO*_x_* layer was successfully synthesized via a pulsed laser deposition method for efficient PEC oxygen evolution.^147^ In addition, Fingerle et al.^148^ took a magnetron‐sputtering method to deposit nickel oxide on the n‐Si/SiO*_x_* photoanode and described the interaction of NiO*_x_* layer and H_2_O at the electrode/electrolyte interface, which was beneficial for the hole transport. All these investigations confirm that nickel oxides can be deposited onto the surface of photoanodes with different methods in order to satisfy various requirements and then gain a better performance.

*Mixed Metal Oxides*: The previous research mainly focused on the functions of monometallic oxide on the photoanodes, while the utilization of mixed metal oxides in PEC‐OER is still under exploration though they usually possess higher conductivity and augmented active surface sites compared with monometallic catalysts. The reasons for that include: 1) mixed metal oxides are hard to grow uniformly onto the surface of semiconductors; 2) the chemical and crystal properties of most mixed metal oxides cannot be compatible with the semiconductors, resulting in the increment of interfacial recombination sites; 3) band alignment between semiconductors/catalysts/electrolytes must be suitable to reduce charge transfer resistance across interfaces; 4) partly due to the lack of predictive models used for the guidance of selecting precursor materials.^149^ Thus, the integration of mixed metal oxides with semiconductors to achieve high efficiency of solar water oxidation is still full of challenge.

**Figure 12:**
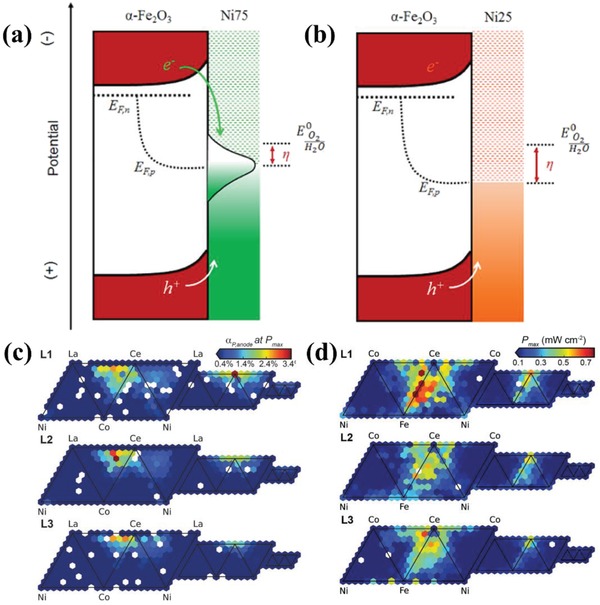
The schematic diagram of the band alignment on the surface of a) Ni _0.75_Fe_0.25_O*_y_* and b) Ni_0.25_Fe_0.75_O*_y_*. Interface trap states exist when coating Ni_0.75_Fe_0.25_O*_y_*. Reproduced with permission.^152^ Copyright 2017, American Chemical Society. c) Composition maps of electrochemical power generation (*P*_max_) for all three discrete composition libraries (L1, L2, L3), Each library contains 286 compositions of Ni–La–Co–Ce oxides. Reproduced with permission.^154^ Copyright 2016, Royal Society of Chemistry. d) Composition maps of electrochemical power generation (*P*_max_) for all three discrete composition libraries (L1, L2, L3), Each library contains 286 compositions of Ni–Fe–Co–Ce oxides. Reproduced with permission.^155^ Copyright 2016, American Chemical Society.

In recent years, some researchers have broken this bottleneck and successfully synthesized the mixed metal oxides coated photoanodes with excellent PEC activity. Wu et al. reported the successful incorporation of NiMoO_4_ nanosheet with TiO_2_/Si nanowire arrays by a facile hydrothermal method for the first time.^149^ This photoanode could achieve a photocurrent value of 8.7 mA cm^−2^ at 2.5 *V*_RHE_, remarkably higher than TiO_2_/Si nanowire arrays. Han et al.^150^ also demonstrated that the CoWO_4_ has very excellent catalytic activity even under mild conditions and can have a nonnegligible effect on the PEC performance of Fe_2_O_3_ photoanode. The existence of CoWO_4_ can cause a significant reduction of charge transfer resistance at the photoanode/electrolyte interface. Thus, the CoWO_4_/Fe_2_O_3_ system exhibits a competitive current density of 1.36 mA cm^−2^, around threefold higher than bare hematite electrode. Furthermore, Xu et al.^151^ and Hajibabaei et al.^152^ reported the binary nickel and iron oxide modified hematite with enhanced efficiency. The performance of Fe‐rich catalyst (Ni_0.25_Fe_0.75_O*_y_*) coated hematite photoelectrode is optimized compared to the bare electrode. While the PEC activity of the Ni‐rich (Ni_0.75_Fe_0.25_O*_y_*) catalyst is lower than the former owing to the presence of interface trap states on the Ni_0.75_Fe_0.25_O*_y_* that serve as the photocarrier recombination centers (**Figure** **12**a,b). These results all indicate that the introduction of bimetallic oxide cocatalysts is capable of remarkably promoting the PEC performances of photoanodes.

Except for bimetallic oxide catalysts, polymetallic oxides like FeNiCoO*_x_* 153 also show great potential for PEC oxygen evolution. Guevarra et al. discovered the BiVO_4_ photoanodes evenly coated with combinatorial integration of Ni–La–Co–Ce oxides,^154^ and studied the properties of 858 mixed metal oxides covering a series of metal oxide loadings and then established a full Ni–La–Co–Ce oxide quaternary composition space. Figure 12c presents the composition maps of electrochemical power generation (*P*_max_) for all three discrete composition libraries (L1, L2, L3), and each library contains 286 compositions of Ni–La–Co–Ce oxides. They found that around one third of the coatings would reduce the reaction efficiency, while selecting suitable combinations of metal oxide composition and loading can realize a topmost increment (14‐fold) for PEC water oxidation in pH 13 electrolytes. Specifically, the antireflection effect of Ce‐rich coatings is beneficial to augment the activity of photoanode, yielding a 20‐fold enhancement in conversion efficiency compared to bare BiVO_4_. Subsequently, they also explored the effects of Ni–Fe–Co–Ce oxides onto BiVO_4_ photoanodes (Figure 12d).^155^ The result demonstrates that a specific combination of metal oxide composition and loading facilitates the yield of oxygen achieving a 13‐fold increment in alkaline conditions. Their work provides a valuable reference for the research of pertinent interfaces and engineering mixed metal oxides anchored photoanodes with high performance for PEC water oxidation.

### 3.3 Metal (Oxy)hydroxides

It is well known that metal (oxy)hydroxides^156^, ^157^ and their derivatives^158^, ^159^ are among the well‐established catalysts for PEC‐OER process due to their low price, natural abundance, and abilities to lead to a large potential drop of Helmholtz layer at the photoanode/electrolyte junction.^160^ Most recently, some researches uncovered that metal (oxy)hydroxides have the potential to serve as the active cocatalysts integrated with suitable photoelectrodes for PEC water oxidation. Among these materials, nickel‐based, iron‐based, cobalt‐based (oxy)hydroxides, as well as layered double hydroxides (LDHs) exhibit the superior performance and gained extensive interest.

#### 3.3.1 Nickel‐Based (Oxy)hydroxides

Numerous researches have confirmed that nickel (oxy)hydroxide cocatalysts can bring about an obvious negative shift of onset potential and a higher PEC water oxidation performance on various semiconductor photoanodes like hematite, vanadate, silicon, and vice versa.^161^, ^162^, ^163^, ^164^, ^165^ Quiñonero and Gómez^163^ coupled a small amount of Ni(OH)_2_ on α‐Fe_2_O_3_ films for improved PEC water oxidation activity. Liu et al. also reported the Ni(OH)_2_‐modified H‐ZnO/CdS photoanode, which could achieve an enhanced photocurrent density of 4.65 mA cm^−2^ at 0.4 *V*_Ag/AgCl_.^166^

**Figure 13:**
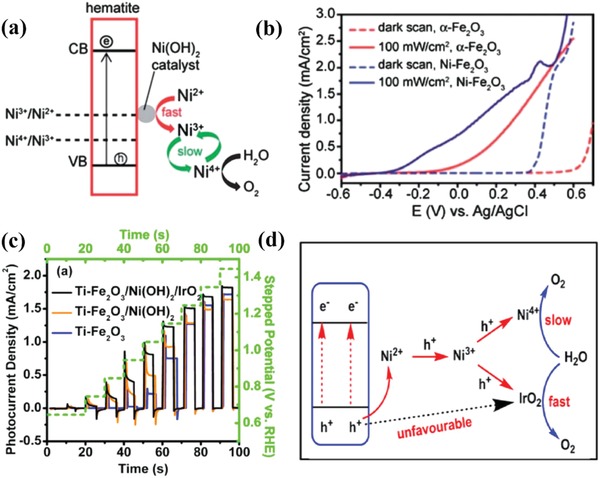
a) Schematic diagram of the proposed catalytic mechanism of Ni(OH)_2_ on hematite for PEC water oxidation. b) *J–V* plots of α‐Fe_2_O_3_ and Ni(OH)_2_/Fe_2_O_3_ in the dark (dashed line) and under light illumination (solid line) in 1.0 m KOH solution at a scan rate of 50 mV s^−1^. Reproduced with permission.^167^ Copyright 2013, Royal Society of Chemistry. c) Photocurrent of Ti‐Fe_2_O_3_ (blue), Ti‐Fe_2_O_3_/Ni(OH)_2_ (black), and Ti‐Fe_2_O_3_/Ni(OH)_2_/IrO_2_ (black) under stepped potential (green, dashed curve). d) Scheme for the charge transfer from hematite to H_2_O through Ni(OH)_2_ and/or IrO_2_. Reproduced with permission.^168^ Copyright 2015, American Chemical Society.

In 2013, Wang et al.^167^ elucidated a detailed mechanism study about the role of Ni(OH)_2_ in PEC water splitting. As shown in **Figure** **13**a, the effects of Ni(OH)_2_ include two steps: first, the Ni^2+^ ions are oxidized to Ni^3+^ ions rapidly; then the Ni^3+^ ions are oxidized to Ni^4+^ ions at a low speed. The achieved Ni^4+^ ions are recognized as the actual active centers for water oxidation. However, the accumulated Ni^3+^ ions slowly formed Ni^4+^ ions and their instability significantly limited the application of Ni(OH)_2_, although the introduction of Ni(OH)_2_ can indeed enhance current density (Figure 13b). Since then, strategies have been adopted to promote the activity and stability of Ni(OH)_2_. For example, Wang et al.^168^ coupled Ni(OH)_2_ with more active IrO_2_ to realize its full catalytic activity (Figure 13c). As Figure 13d describes, with the introduction of IrO_2_, another channel to transfer holes is formed. Through this channel, holes can be transformed to IrO_2_ rapidly and then consumed for the water oxidation rather than oxidizing Ni^3+^ species to higher oxidation state, which largely enhanced the rate of water oxidation. He et al.^164^ replaced the pure Ni(OH)_2_ with flagella nanowire‐modified Ni(OH)_2_ (F‐Ni(OH)_2_) on Fe_2_O_3_ photoanode. It reveals that the addition of flagella nanowires could accelerate the interface transfer of holes, reduce the hole accumulation, and then improve the catalytic activity of Ni(OH)_2_.

Similarly, NiOOH also possesses good stability and impressive PEC water splitting catalytic performance compared with Ni(OH)_2_. For instance, the NiOOH coated (Sn, Zr) codoped Fe_2_O_3_ photoanode demonstrated by Tamirat et al. gives rise to a reduced onset potential of 0.58 V and an improved photocurrent density of 1.64 mA cm^−2^ at 1.23 *V*_RHE_.^169^ And a Si/SiO*_x_*/ITO/NiOOH hybrid photoanode^170^ was also revealed to provide plentiful active sites, facilitating the water oxidation efficiency to a large extent. All these works indicate practical applications of NiOOH for solar driven water oxidation. However, one of the defects restricting the application of NiOOH is that up to now the only valid synthesis route to synthesize NiOOH is the photoassisted electrodeposition.^161^ Therefore, an alternate deposition method of NiOOH is still highly desired.

#### 3.3.2 Iron‐Based Oxyhydroxide

Iron‐based oxyhydroxide has also been extensively investigated for efficient PEC oxygen production.^33^, ^73^, ^101^, ^171^, ^172^, ^173^, ^174^, ^175^, ^176^ It has potential advantages such as feasible synthesis, well stability, and resource abundancy.^173^, ^176^ Yu et al.^176^ reported the synthesis Fe_2_O_3_/FeOOH photoanode for better PEC water oxidation. Gong and co‐workers^171^ reported a FeOOH‐loaded TiO_2_/Ti:Fe_2_O_3_ photoanode. With the introduction of FeOOH, the charge separation efficiency on the surface is significantly increased to 90% at 1.23 *V*_RHE_.

**Figure 14:**
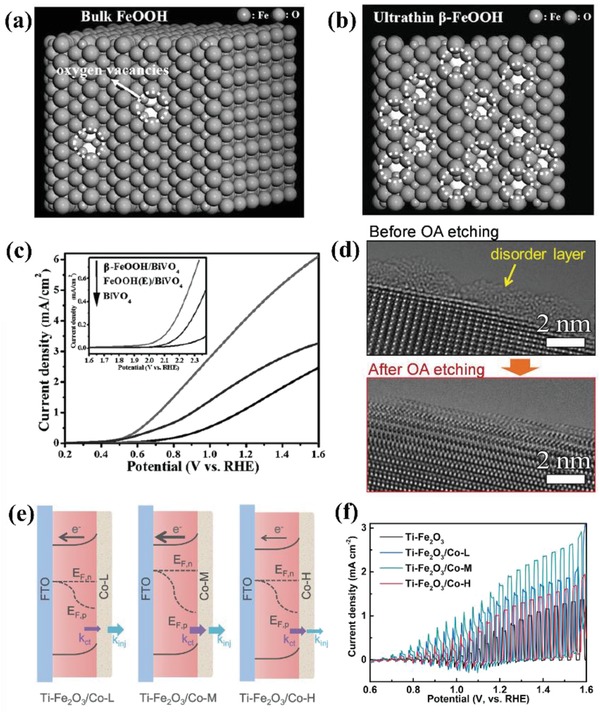
Crystal models of a) bulk FeOOH and b) ultrathin FeOOH with different oxygen vacancies. c) *J–V* curves of β‐FeOOH/BiVO_4_ photoanodes measured with 0.2 m Na_2_SO_4_ with and without (inset) light under AM 1.5G (100 mW cm^−2^). Reproduced with permission.^73^ Copyright 2018, Wiley‐VCH. d) HR‐TEM images of Ti/flame‐d hematite NRs before (top) and after (bottom) the OA etching. Reproduced with permission.^33^ Copyright 2015, Wiley‐VCH. e) Interfacial energetics of Co‐based cocatalyst coated Ti‐Fe_2_O_3_ photoanodes under illumination. The photogenerated electrons (*e^−^*) are collected by the FTO substrate, and the holes go through the charge transfer and injection into electrolyte for water oxidation. *V*_ph_ is associated with the quasi‐Fermi level of holes (*E*_F_,_p_) and electrons (*E*_F_,_n_). The wider arrow means a faster charge‐transfer rate. f) Chopped *J–V* plots of Ti‐Fe_2_O_3_ coupled with Co‐L (blue), Co‐M (green), or Co‐H (red) under light. Reproduced with permission.^178^ Copyright 2018, American Chemical Society.

However, with further investigations, some inherent limitations such as large thickness, poor electrical conductivity, and low hole transfer kinetics, which greatly restrict their further application, have been gradually discovered. Recently, some innovative strategies have been taken to address these issues.^33^, ^73^, ^101^, ^173^, ^175^ A novel method of solution impregnation was utilized by Zhang et al.^73^ to synthesize oxygen vacancies enriched β‐FeOOH ultrathin nanolayers on BiVO_4_. The morphology differences of bulk FeOOH and this β‐FeOOH can be seen in **Figure** **14**a,b separately. Finally, the designed complex photoanode achieved a high photocurrent density of 4.3 mA cm^−2^ at 1.23 *V*_RHE_, around twofold of the amorphous FeOOH nanofilms fabricated by electrodeposition (Figure 14c). Cho et al.^33^ innovatively added an extra oxalic acid etching step before depositing FeOOH onto Fe_2_O_3_, as Figure 14d presents, which could get rid of the surface disorder layer of hematite nanorods (NRs), enhance charge transfer efficiency, and largely improve the interface quality between the OER cocatalyst and photoanode.

#### 3.3.3 Co‐Based (Oxy)hydroxides

Moreover, Co‐based (oxy)hydroxides were also recognized as the robust and promising cocatalysts to lower onset potential and favor injection of charge carriers.^177^ Many groups have been working on it.^103^, ^177^, ^178^, ^179^, ^180^, ^181^, ^182^ A Co(OH)*_x_* cocatalyst could help Ta_3_N_5_ photoanode have a significantly improved photocurrent of 5.5 mA cm^−2^ at 1.23 *V*_RHE_, which is one of the highest values among all currently available Ta_3_N_5_ photoanodes.^182^ And Tang et al.^103^ demonstrated that the coating of CoOOH onto BiVO_4_ could form oxobridge bonding which can remove the surface trapping states and enhance photocurrent.

Lately, some researchers have explored the intrinsic factors that affect catalytic activity of Co‐based (oxy)hydroxides. For example, Xu et al.^178^ described a successful synthesis of Ti‐doped Fe_2_O_3_ photoanode coated with CoOOH of three different crystallinities for water oxidation. They found that the moderate crystalline CoOOH can partially screen electrolyte, thus leading to an ideal band bending for charge separation and the most highly activity for water oxidation among these three kinds of CoOOH with various crystallinity. The interfacial energies and photocurrent when coated with CoOOH of different crystallinity are presented in Figure 14e,f. Wang et al.^180^ utilized a two‐step impregnation method to load untrasmall CoO(OH)*_x_* nanoparticles onto TiO_2_ and polyheptazine (TiO_2_–PH) hybrid photoanodes for water oxidation, the photocurrent of which remarkably outperforms that of conventional cocatalyst Co–Pi. They emphasized that the structural and optical properties will both have an impact on the activity of cocatalysts.

#### 3.3.4 LDHs

**Figure 15:**
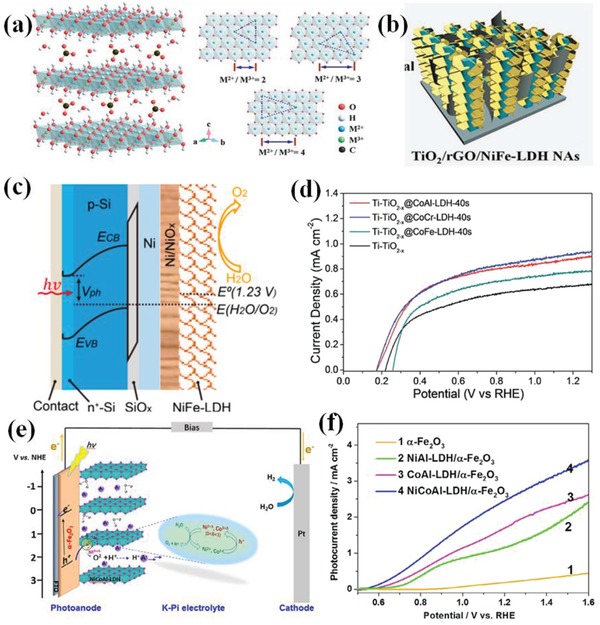
a) The structure model of carbonate‐intercalated LDHs with different M^2+^/M^3+^ molar ratios showing the metal hydroxide octahedra stacked along the crystallographic *c*‐axis. Water and anions are present between the interlayers. Reproduced with permission.^186^ Copyright 2014, Royal Society of Chemistry. b) The morphology model of TiO_2_/rGO/NiFe‐LDH system. Reproduced with permission.^195^ Copyright 2016, Royal Society of Chemistry. c) Cross‐sectional schematic and energy band structure of n^+^p‐Si/SiO*_x_*/Ni/NiO*_x_*/NiFe‐LDH photoanode for water oxidation. Reproduced with permission.^196^ Copyright 2018, American Chemical Society. d) *J–V* curves of Ti‐TiO_2‐_ *_x_*@LDH deposited for 40 s with three kinds of LDH (CoAl‐LDH, CoCr‐LDH, and CoFe‐LDH) under illumination. Reproduced with permission.^201^ Copyright 2017, Royal Society of Chemistry. e) The proposed mechanism of water splitting on NiCoAl‐LDH coated α‐Fe_2_O_3_ photoanode and Pt cathode. f) LSV curves of α‐Fe_2_O_3_, CoAl‐LDH/α‐Fe_2_O_3_, NiAl‐LDH/α‐Fe_2_O_3_, and NiCoAl‐LDH/α‐Fe_2_O_3_ under AM 1.5G radiation in the 0.5 m K‐Pi solution (pH 7) with a scan rate of 20 mV s^−1^. Reproduced with permission.^48^ Copyright 2018, Elsevier.

LDHs, usually represented by the chemical formula [M_1−_ *_x_* ^2+^M*_x_* ^3+^(OH)_2_]*^x^* ^+^[A*_x/n_*]^n−^·mH_2_O,^183^, ^184^, ^185^ belong to a large family of anionic clay materials and can be characterized by hydrotalcite‐like layered structure, tunable composition, as well as flexible ion exchangeability. The lamellar framework (**Figure** **15**a) consists of evenly distributed divalent and trivalent metal cations, with anions intercalated.^186^ This structure avails fast diffusion of reagents and products and efficient proton‐coupled electron transfer process, making them become excellent catalyst candidates for PEC‐OER.^187^, ^188^

Lately, some 3D earth‐abundant metal‐based LDHs have been successfully synthesized. It is proved that they are outstanding cocatalysts of PEC water oxidation and can significantly enhance the yield of oxygen. Nowadays, the most commonly used LDH is NiFe‐LDH, as Ni and Fe are both earth‐abundant and it shows the best catalytic activity among the Ni‐based LDH, which was reported by Diaz‐Morales et al.^189^ They have been applied to some potential metal‐based photoanodes including Ta_3_N_5_,^190^ BiVO_4_,^191^, ^192^ WO_3_,^193^ α‐Fe_2_O_3_,^194^ and have shown promoted performance in onset potentials, photocurrent densities, as well as photostability. Other researchers incorporated nonmetal compounds like graphene^188^, ^195^ or carbon dots^187^ with NiFe‐LDH, and found their synergetic effect could further propel the PEC water oxidation process. Taking the TiO_2_/rGO/NiFe‐LDH system^195^ (Figure 15b), for example, the high work function and superior electron mobility of rGO contribute to the fast electron transportation and the introduction of NiFe‐LDH enables faster surface water oxidation reaction kinetics. In this system, a high photocurrent density of 1.74 mA cm^−2^ at 0.6 *V*_RHE_ is achieved, which outdistanced the TiO_2_‐based photoanodes once reported in benign and neutral media. Besides, Guo et al.^196^ reported a special bridging layer strategy to overcome the bottleneck that LDHs are difficult to be coupled with Si‐based photoelectrodes. Figure 15c depicts the Ni/NiO*_x_* interlayer simultaneously contacting photoabsorber Si and NiFe‐LDH nanosheet array. The result showed that this system could afford a very low onset potential (≈0.78 *V*_RHE_) and a high photocurrent density (≈37 mA cm^−2^ at 1.23 *V*_RHE_) maintaining for 68 h in alkaline conditions under AM 1.5G. It is the highest OER activity reported to date for the crystalline Si‐based photoanodes.

Except for NiFe‐LDHs, other metal‐based LDHs like CoNi‐LDH,^75^, ^197^, ^198^ CoAl‐LDH,^199^, ^200^, ^201^ ZnFe‐LDH,^74^ etc., also emerge high PEC‐OER performance. For example, α‐Fe_2_O_3_ photoelectrode coupled with CoAl‐LDH was fabricated by Chong et al.^199^ It assisted the α‐Fe_2_O_3_ photoanode to remain remarkably stable in near neutral electrolyte and simultaneously showed outstanding activity due to the synergistic effect between Co^2+^, providing active catalytic sites for OER, and Al^3+^, giving support for the layered skeleton. The result helps further investigations of PEC water oxidation in neutral pH environments. Based on the previous research, Guo et al.^201^ compared the different catalytic activities between three common LDH materials, including CoAl‐LDH, CoCr‐LDH, and CoFe‐LDH. They employed them to incorporate with reduced titania for solar water oxidation and found that the reduced titania@CoCr‐LDH exhibited the highest efficiency for PEC oxygen evolution, as Figure 15d shows. The result reveals that a good match of the band structure helps boost driving force and the migration of holes from reduced titania to LDH, followed by the LDH catalyzing water oxidation.

Whereas, though Co‐ or Ni‐based binary‐component LDHs have shown enhanced PEC water oxidation performance, they still suffered from low electrical conductivity because of the fully occupied bonding *t*_2g_ orbitals of MO_6_ center.^202^ Recent researches revealed that ternary‐component LDHs containing three kinds of transition metals could realize faster migration of photocarriers and higher conductivity.^203^, ^204^, ^205^ Therefore, engineering photoanodes with ternary‐component LDHs is probably a good choice to further boost PEC water oxidation efficiency. For example, a α‐Fe_2_O_3_ photoanode modified by ultrathin NiCoAl‐LDH via in situ growth was reported to show superior efficiency and stability in neutral pH electrolytes (Figure 15e).^48^ The onset potential shown in Figure 15f is 0.55 *V*_RHE_ and the photocurrent density is 2.56 mA cm^−2^ at 1.23 *V*_RHE_, which is 13, 2, and 1.4 times higher than that of pure α‐Fe_2_O_3_, NiAl‐LDH/α‐Fe_2_O_3_, and CoAl‐LDH/α‐Fe_2_O_3_, respectively.

### 3.4 Metal‐Based Boron Compounds

**Figure 16:**
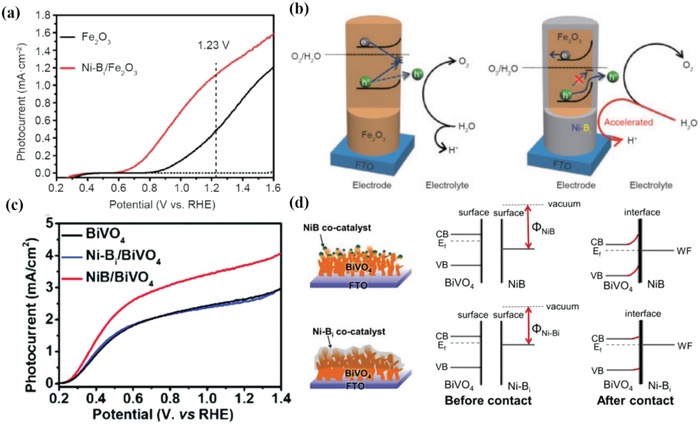
a) *J–V* curves of Fe_2_O_3_ with and without Ni–Bi under AM 1.5G illumination at 0.5 m KBi electrolyte (pH 9.2). b) Schematic illustration of charge transfer before (left) and after (right) adding Ni–Bi cocatalyst on the Fe_2_O_3_ photoanode.^210^ c) *J–V* curves of bare BiVO_4_, Ni–Bi coated BiVO_4_, and NiB coated BiVO_4_ under AM 1.5G in 1 m Na_2_SO_3_. d) Schematic of NiB and Ni–Bi‐decorated BiVO_4_ and the mechanism of charge separation. Reproduced with permission.^216^ Copyright 2017, Royal Society of Chemistry.

To date, some studies have shown the tremendous promotion of PEC‐OER performance catalyzed by nickel‐borate (Ni–Bi)^206^, ^207^ and cobalt‐borate (Co–Bi).^208^ As the derivatives of Co–Pi, they share the similar catalytic mechanisms for facilitating PEC water oxidation process.^209^ Besides, these nonprecious catalysts in alkaline environments exhibit competitive activity and stability with IrO_2_ and Co–Pi.^210^ Hence, it can be speculated that these metal‐borate catalysts can also be used as very promising cocatalysts for higher PEC water oxidation efficiency. Hong et al.^211^ reported the introduction of Ni–Bi onto the Fe_2_O_3_, which is the first time that Ni–Bi was deposited onto a photoanode for water oxidation to the best of our knowledge. The obtained photoanode brought about a large cathodic shift of the onset potential and enhanced photocurrent, providing a large inspiration for deeper research. After that, Choi et al.^209^ integrated NiBi with BiVO_4_ via photodeposition and electrodeposition, and they revealed the catalytic activity of Ni–Bi largely depended on the deposition methods and time. Gan et al.^212^ reported that the Ni–Bi layer on the BiVO_4_ photoanode enables a 350 mV cathodic shift of onset potential for PEC water oxidation at pH 9. Though the study on Ni–Bi had made some progress, the role of Ni–Bi as a cocatalyst is still not completely explicit in PEC water oxidation process. However very recently, Dang et al.^210^ reported the Ni–Bi/Fe_2_O_3_ photoanode with enhanced PEC capability (**Figure** **16**a) and demonstrated two functions of Ni–Bi in this system: Ni–Bi can unpin the Fermi level and lead to an enhanced upward band bending. Specifically, The Bi in the Ni–Bi facilitates releasing protons and then prompts the PEC performance. The schematic diagram of mechanism is shown in Figure 16b. His elaboration may shed more lights on our understanding of the mechanism of Ni–Bi.

Cobalt borate (Co–Bi) is another robust cocatalyst which has been reported to be applied in PEC‐OER.^213^, ^214^ A recent research has also reported that the performance of Co–Bi cocatalysts photodeposited on SrTiO_3_ photoelectrodes for water oxidation is superior to Co–Pi under the same conditions due to that the concentration of Co–Bi on SrTiO_3_ is higher than that of Co–Pi. This indicates there are much more reaction sites on the Co–Bi/ SrTiO_3_ compared with Co–Pi/ SrTiO_3_.^215^

In addition, transition‐metal borides such as NiB alloy which possess the metallic nature and the significant electron interaction between metal and B also belong to the family of boron compounds, but the activity is quite distinguished from the transition‐metal borate cocatalyst. For instance, Dang et al.^216^ demonstrated the difference between NiB cocatalyst and Ni–Bi cocatalyst coupled with BiVO_4_. It is found that the loading of the NiB can lead to a lower onset potential and a higher photocurrent (Figure 16c) due to a more conspicuous upward band bending compared with the loading of the Ni–Bi cocatalyst. They explained that the higher work function of NiB can be responsible for this phenomenon (Figure 16d). In fact, much attention has been paid to transition‐metal borate catalyst, while transition‐metal borides applied in water splitting are rarely investigated though they are very common in electrocatalysis. Hence, it is still necessary for us to explore more potential of these transition‐metal borides for PEC water oxidation process.

### 3.5 Metal‐Free Cocatalysts

It can never be ignored that usually the materials containing metallic ions tend to be toxic, which could limit their further applications. Besides, the large thicknesses or dimensions of these materials probably hinder sunlight absorption and prolong the transfer distances of photogenerated holes.^217^ Hence, it would be an alternative choice for researchers to work on the investigation of novel metal‐free cocatalysts for favoring the solar water oxidation.^218^

Graphene is a very promising metal‐free material which contains 2D sp^2^ carbon atoms aligned in a honeycomb structure and has been widely incorporated with semiconductors owing to its intrinsic physicochemical properties and large specific surface area.^219^ Lately, graphene has been introduced in some PEC system in order to facilitate water oxidation. For example, Ahmed et al.^220^ fabricated TiO_2_ photoanodes coupled with graphene for solar fuel generation. In this process, graphene works as an electron transfer channel and accelerates the kinetics of electron transportation, leading water oxidation reaction to be much more efficient.

**Figure 17:**
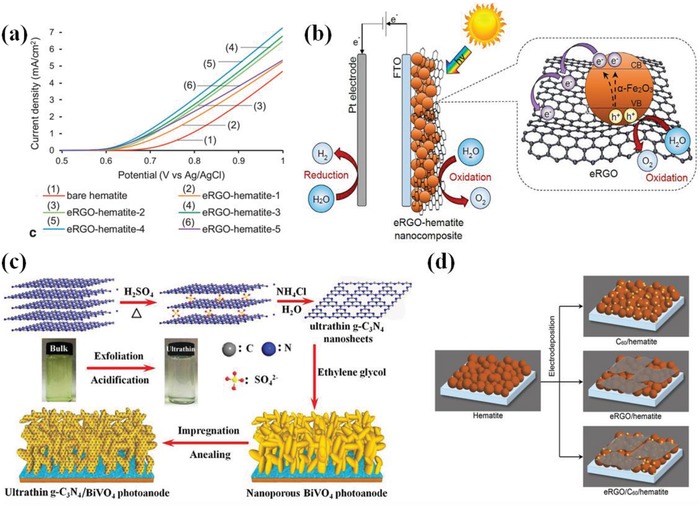
a) *J–V* curves of the bare hematite and eRGO hematite‐i nanocomposites. b) Schematic of charge transfer in the eRGO‐hematite nanocomposite for water splitting. Reproduced with permission.^222^ Copyright 2017, Elsevier. c) Schematic illuminating of the exfoliation and acidification process for fabricating ultrathin g‐C_3_N_4_‐NS and BiVO_4_/g‐C_3_N_4_‐NS photoanodes. Reproduced with permission.^217^ Copyright 2017, Elsevier. d) Schematic illustration of electrodeposition process of hematite nanocomposites photoanodes. Reproduced with permission.^234^ Copyright 2017, Wiley‐VCH.

One of the derivatives of graphene, denoted as reduced graphene oxide (rGO), has the similar properties with graphene but higher conductivity, thus also used as a PEC‐OER cocatalyst. Zhang et al.^221^ reported that the rGO integrated on ITO/hematite nanowires could remarkably boost the water oxidation. The complex photoanode could yield a photocurrent of 5.38 mA cm^−2^ at 0.6 V versus saturated calomel electrode (*V*_SCE_), a 3.5‐fold and tenfold of ITO/Fe_2_O_3_ and pure Fe_2_O_3_ photoanode respectively under same circumstances. Phuan et al.^222^ designed an e‐rGO (electrochemical reduced graphene oxide)/hematite photoanode via a two‐step electrodeposition method with high PEC performance. The existence of e‐rGO brings about an eightfold increment of photocurrent density compared with bare hematite (**Figure** **17**a), which can be ascribed to the enhanced light absorption, added active reaction sites, and accelerated electron transfer. The schematic diagram explaining how the cocatalyst works is presented in Figure 17b. Other studies^223^, ^224^ also imply that rGO can improve the conductivity of the surface and eventually give rise to an enhanced PEC performance. In order to further promote the catalytic activity and durability of rGO, heteroatom doping strategy has been taken. Common heterodopants include nitrogen, phosphorous, and sulfur.^225^, ^226^, ^227^ Part of the carbon sites in the framework structure of rGO can be replaced with these heterodopants and the introduction of defects also arises from them. Elbakkay et al.^219^ successfully synthesized the S‐TiO_2_/S‐rGO photoanodes. They found that the doping of S could make the thickness of rGO cut down to 0.51 nm and the roughness risen up to 1.328 mm, resulting in a higher photocurrent density of 3.36 mA cm^−2^ at 1 *V*_Ag/AgCl_ than the pure TiO_2_ photoanode.

On top of abovementioned materials, graphitic carbon nitride (g‐C_3_N_4_) with a structure of tri‐s‐triazine/heptazine also gives rise to a new research upsurge among researchers due to its high performance in the field of energy conversion recently.^228^ Feng et al.^217^ elucidated a simple ethylene glycol dispersion and impregnation method to fabricate ultrathin graphitic C_3_N_4_ nanosheets (g‐C_3_N_4_‐NS) coated BiVO_4_ photoanodes, as can be seen in Figure 17c. These ultrathin g‐C_3_N_4_ nanolayers can not only restrain the interfacial charge recombination of BiVO_4_, but also promote the transformation and storage of photogenerated holes for water oxidation. In addition, Huang et al.^229^ reported the N‐Doped carbon dots acting as the cocatalyst on Fe_2_O_3_. Under simulated solar irradiation, the photocurrent density of N‐CDs@α‐Fe_2_O_3_/Ti photoanode shows a 3.4‐fold increment with a 280 mV negative shift of the onset potential and better durability compared with that of pristine α‐Fe_2_O_3_/Ti. Other common carbonaceous materials that can be employed as cocatalysts also include CNT,^230^, ^231^ fullerene,^232^, ^233^ etc. Very recently, a novel ternary hematite photoanode with fullerene and 2D‐electrochemical rGO was designed by Phuan et al.,^234^ as shown in Figure 17d, the introduction of C60 and rGO contributes to a 16.8‐fold enhancement in photocurrent density and a 0.8‐fold reduction in charge transfer resistance in comparison with the pure hematite photoanode.

Generally, all the above results confirm that metal‐free materials are the competitive cocatalysts for further application and play a pivotal role in boosting the PEC performance. Hence, it can be expected that metal‐free cocatalysts can provide a different route to develop more photoanodes with higher performance for oxygen evolution.

### 3.6 Non‐Noble Metal Atoms

**Figure 18:**
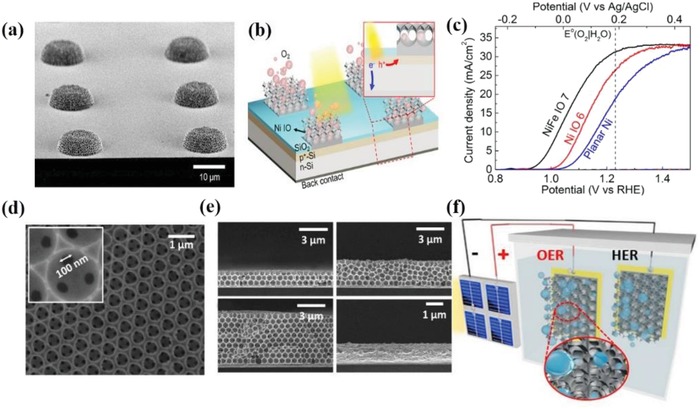
a) Tilted‐view SEM image of oxide‐passivated p^+^n‐Si photoanode with the micropatterned Ni IOs. b) Schematic illustrating of water oxidation on Ni IO coated Si‐based photoanode. Inset is the charge transfer inside the photoanode after irradiation. c) PEC *J–V* curves of the Si‐based photoanode integrated with micropatterned planar Ni (blue line), NiIO_6_ (red line), and NiFeIO_7_ (black line). Reproduced with permission.^245^ Copyright 2017, American Chemical Society. d) Top scanning electron microscope (SEM) images of NiFe inverse opal (IO) structures. The inset shows mass transport channels with a diameter of ≈100 nm. e) Cross‐section of NiFeIO structures with 2.5 (above left), 5 (above right), and 10 thickness layers (below left) and NiFe planar film for comparison (below right). f) Scheme of the electrolyzer (EZ)‐photovoltaic (PV) combined system for overall water oxidation. Reproduced with permission.^246^ Copyright 2017, Elsevier.

Selecting non‐noble metals such as Ni, Fe, Bi, etc., to be substitutes of precious metal‐based cocatalysts to improve solar water oxidation is also a good choice, as they are earth‐abundant, low‐cost, and recently reported to be the excellent electrocatalysts,^235^, ^236^, ^237^ with decent OER performance in basic conditions. Ueda et al.^238^ reported the Co modified BaTaO_2_N photoanode. It achieved a photocurrent of 4.2 mA cm^−2^ at 1.2 *V*_RHE_ under AM 1.5G and a STH efficiency of 0.7% at 1.0 *V*_RHE_. Wang et al.^239^ deposited Bi film onto BiVO_4_ via photoassisted reduction. They adopted the large work function of Bi particles and its ability to capture electrons to generate a broader visible light absorption edge and enhanced charge separation. Furthermore, nickel,^240^, ^241^, ^242^, ^243^, ^244^ as a member of non‐noble metal family, is capable of resisting photocorrosion and providing high OER activity. Its modification is considered as a facile and efficient route to enhance water oxidation, thus it has also been widely investigated. Lately, Oh et al.^245^ have introduced a novel photo‐electrochemical system with Si‐based photoanode in alkaline solutions. They integrated the photoanode with an orderly 3D porous nanostructure, defined as Ni inverse opal (IO) (**Figure** **18**a), in order to tackle the limited water oxidation efficiency arising from the curbed surface area of conventional cocatalysts coated onto photoanodes (Figure 18b). By using the Ni IOs to enlarge surface area of cocatalyst and hence offering more active sites, the overpotential is reduced by 120 mV compared with planer Ni coupled Si photoanode (Figure 18c). After that, they synthesized the NiFe alloy IOs for solar water oxidation. The top and cross‐section of scanning electron microscope (SEM) images of NiFe IO structures and the IO structures with 2.5, 5, and 10 thickness layers as well as NiFe planar film can be seen in Figure 18d,e. The onset potential is reduced to 0.94 *V*_RHE_ and the photocurrent density rises up to 31.2 mA cm^−2^ at 1.23 *V*_RHE_ in alkaline environment under 1 sun irradiation.

In the same year, Song et al.^246^ also reported the NiFe IOs which can serve as bifunctional cocatalysts incorporated to Si‐based photoanode (Figure 18f). Ultimately, this system realized the overall water splitting and resulted in a 9.54% STH efficiency after 24 h without bias, very close to the requirement of commercial utilization. In a word, this special 3D nanostructure brings significant benefit in PEC water oxidation and it is believable that these investigations can undoubtedly inspire researchers to design more distinguished bifunctional cocatalysts for solar energy conversion.

### 3.7 Molecular Cocatalysts

**Figure 19:**
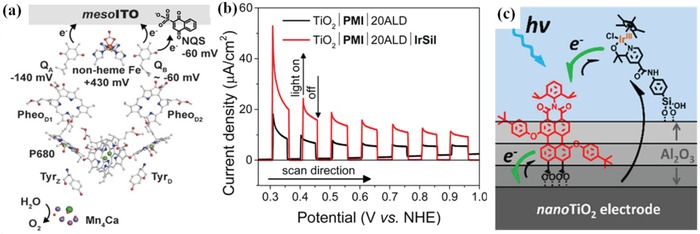
a) Schematic representation of an electron transfer chain in mesoITO and cocatalysts. Reproduced with permission.^248^ Copyright 2015, American Chemical Society. b) Chopped LSV curves of TiO_2_|PMI|20ALD|IrSil without (black) and with (red) catalyst in 0.1 m KNO_3_ at pH 5.8. c) The schematic diagram of TiO_2_|dye|Al_2_O_3_|IrSil electrodes. Reproduced with permission.^253^ Copyright 2017, American Chemical Society.

Molecular materials have also been assembled onto photoanode as cocatalysts for PEC‐OER due to their large surface area and porous structure. Enlightened by the role of enzyme Photosystem II (PSII) for water oxidation in natural photosynthesis, researchers attempt to design PSII coated photoanodes for artificial PEC water oxidation.^247^ For example, Kato et al.^248^ reported a complex photoanode consisting of PSII and a mesoporous indium‐tin oxide (meso‐ITO) electrode which is 3D and biocompatible for solar water oxidation (**Figure** **19**a). This employed PSII‐meso‐ITO photoanode allows for a direct electron transfer from meso‐ITO to PSII. Others also reported the formation of entire solar‐driven water splitting devices consisted of PSII anchored photoanodes and photocathodes^249^, ^250^, ^251^ and these cells also achieved a desired STH efficiency. Except PSII, other metal‐based molecular catalysts also come into sight. It is reported that a Co‐based molecular catalyst was coated onto an amorphous silicon photoanode which carried out a water splitting reaction efficiency of 4.7% for a wired configuration and 2.5% for a wireless configuration under 1 sun.^252^ Kamire et al.^253^ also demonstrated that the organic dye‐sensitized nano‐TiO_2_ photoanode coated with IrSil molecular catalyst could enhance photocurrent and reduce charges recombination. Figure 19b,c shows the promoted photocurrent density after introducing IrSil cocatalyst and its schematic diagram, respectively. However, many metal‐based molecular catalysts will gradually become unstable over time due to the photodegradation.^17^ Therefore, tackling this shortcoming is indispensable if we want to further investigate their functions as OER cocatalysts.

### 3.8 Dual Cocatalysts

**Figure 20:**
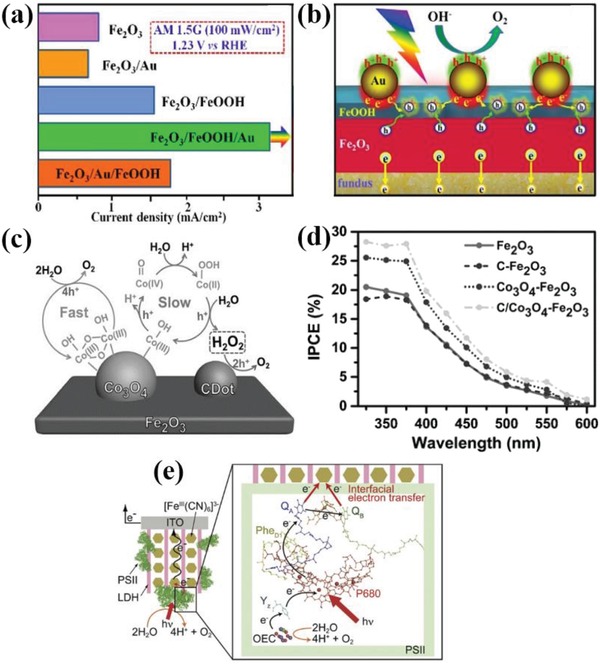
a) Comparison of photocurrent densities at 1.23 *V*_RHE_ for various photoanodes. b) Schematic illumination of the charge transfer in “hole‐depletion” layer of α‐Fe_2_O_3_/FeOOH/Au NFs. Reproduced with permission.^173^ Copyright 2017, Elsevier. c) Schematic illustration of the fast and slow reaction processes on Co_3_O_4_ cocatalyst and the two‐step‐two‐electron reaction pathway for water oxidation on the CDs/Co_3_O_4_‐Fe_2_O_3_ photoanode. d) IPCE (%) of Fe_2_O_3_, C‐Fe_2_O_3_, Co_3_O_4_‐Fe_2_O_3_, and C/Co_3_O_4_‐Fe_2_O_3_ photoanodes. Reproduced with permission.^258^ Copyright 2016, Wiley‐VCH. e) Schematic representation of the oxygen production initiated by MAl_e_[Fe(CN)_6_]|PSII (M = Mg or Co) photoanodes under visible light. Reproduced with permission.^261^ Copyright 2017, Elsevier.

Thus far, most related studies concentrate on the roles of single cocatalysts for PEC water oxidation, while few about dual cocatalysts have been reported. However, compared with single cocatalysts, dual cocatalysts^195^, ^254^, ^255^, ^256^, ^257^ tend to possess different functions, hence they usually emerge superiorities of higher PEC water oxidation performance due to synergetic effects caused by their interactions.^258^ For example, it is reported that a FeOOH and Au dual cocatalytic system helps α‐Fe_2_O_3_ become one of the photoanodes with highest PEC performance ever reported for water oxidation (**Figure** **20**a).^173^ In this system, FeOOH is responsible for trapping holes and then carrying them to the interface efficiently, while the Au NPs give electrons to neutralize holes, allowing water oxidation reaction to proceed sustainably at the interface of α‐Fe_2_O_3_/electrolyte (Figure 20b). In addition, novel Co_3_O_4_/Co–Pi nanosheets with mesoporous and networked structure were synthesized in a simple and scalable way.^259^ This kind of hybrid cocatalyst hosts enriched active sites and enlarged surface area, facilitating BiVO_4_ photoanode to achieve an excellent PEC OER performance.

Except the combination of metal‐based cocatalysts, some researchers also synthesized the dual cocatalysts consisting of metal‐based and metal‐free materials. For example, Zhang et al.^258^ conjugated Co_3_O_4_ cocatalyst with graphitic carbon nanodots (CDots) to form a synergetic system and applied this system onto hematite photoanode (Figure 20c). They reported that this system could achieve a 60 mV negatively shifted onset potential, an obvious increment in terms of IPCE values (Figure 20d) and a photocurrent density of 1.48 mA cm^−2^ at 1.23 *V*_RHE_, which is 78% higher than bare hematite photoanode. Besides, rGO/phosphate and rGO/Ni:FeOOH reported by Sun et al.^260^ and Zhang et al.^224^ respectively also show desirable stability and prominently promotive PEC water oxidation capability.

As the molecular materials are gradually applied into PEC process, a few dual cocatalysts composed by organic and inorganic materials have also been successfully designed. Kato et al.^261^ deposited PSII/Ferricyanide‐intercalated LDHs onto the ITO substrates (Figure 20e) and concluded that the photoanode could exhibit a 0.5 ± 0.1 s^−1^ of TOF (turnover frequency) and a 920 ± 40 of TON (turnover numbers) for 1 h, implying it will be a potential material to boost PEC water oxidation. Certainly, all of these studies prove that constructing effective dual cocatalytic systems will be a feasible method to help initiate a new route for developing more outstanding water‐splitting photoanodes with high performance.

### 3.9 Others

**Figure 21:**
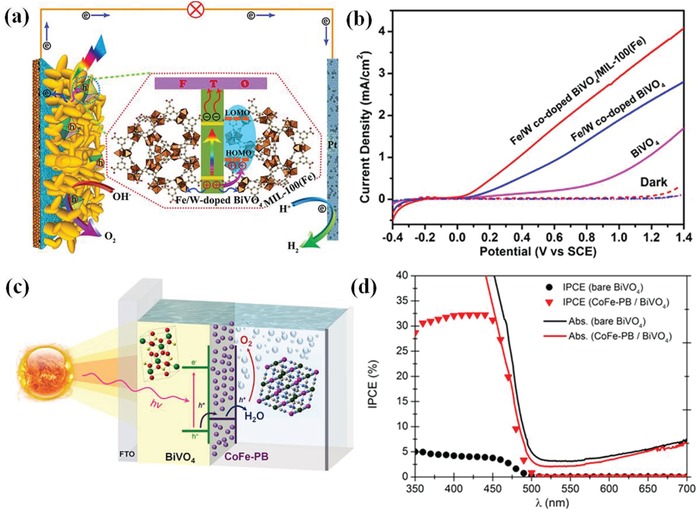
a) Illustration of the charge separation and transfer in Fe/W codoped BiVO_4_ coupled with MIL‐100(Fe). b) *J–t* curves of pure BiVO_4_, Fe/W codoped BiVO_4_, and MIL‐100 (Fe) coated BiVO_4_ under 1 sun irradiation. Reproduced with permission.^262^ Copyright 2016, Wiley‐VCH. c) Schematic diagram of BiVO_4_/CoFe‐PB applied for PEC water oxidation. d) IPCE (%) at 1.23 *V*_RHE_ for bare BiVO_4_ (black circles) and CoFe‐PB‐coated BiVO_4_ (red triangles) at pH 7. Reproduced with permission.^263^ Copyright 2017, American Chemical Society.

On top of above common and deeply investigated cocatalysts, other newly developed cocatalysts such as iron titanate, MOFs, metal carbonate hydroxide, etc., are also recognized as potential cocatalysts but received less attention in prior work. MOFs, which have characteristics of large porosity and high surface area, are usually applied in electrocatalysis, while they are hardly utilized as PEC water oxidation cocatalysts before. Very lately, Tang et al.^141^ fabricated the Co_3_O_4_ cocatalyst derived from MOFs and incorporated it with TiO_2_/Si heterojunction. They reported that the MOF‐derived Co_3_O_4_ could improve higher reaction activity by broadening light absorption range and enriching reaction sites. Jiao et al.^262^ also described a Fe/W Co‐doped BiVO_4_ photoanode loaded with MOF MIL‐100 (Fe) with good activity (**Figure** **21**a). This composite photoanode could lead to a 14‐fold increment of photocurrent density compared with bare BiVO_4_ (Figure 21b), indicating the introduction of MOF will be beneficial for PEC oxygen production.

Besides, Hegner et al.^263^ deposited CoFe prussian blue‐type cocatalysts (CoFe‐PB) onto BiVO_4_ photoelectrode (Figure 21c), resulting in a 6 times increment of IPCE at the same wavelength and a decrease of ≈15% of stability over 50 h irradiation (Figure 21d). Cao et al.^264^ also demonstrated ZnNiCo carbonate hydroxide (ZNC‐CH) anchored TiO_2_ nanorod arrays, exhibiting high oxygen evolution performance with photocurrent reaching 1.16 mA cm^−2^ at 1.23 *V*_RHE_ and onset potential negatively shifted from 690 to 620 mV. It is noteworthy that there is still a sea of undeveloped cocatalysts with remarkable properties remaining to be investigated, hence unremitting efforts need to be devoted to it in order to realize a much higher efficiency of PEC water oxidation.

### 3.10 Factors Affecting Catalytic Activities of Cocatalysts

**Figure 22:**
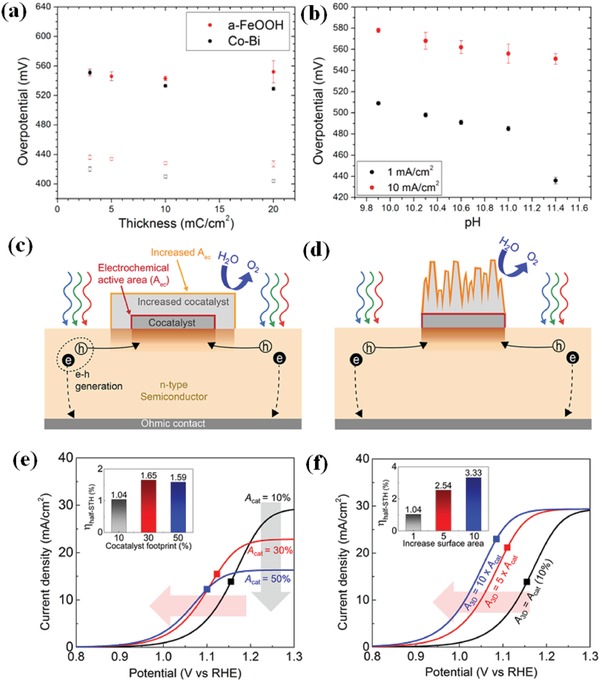
a) Overpotential versus thickness at 1 mA cm^−2^ (hollow circles) and 10 mA cm^−2^ (filled circles) for α‐FeOOH and Co‐Bi in 1 m Na_2_CO_3_. b) Overpotential versus pH at 1 mA cm^−2^ (black circles) and 10 mA cm^−2^ (red circles) for α‐FeOOH and Co‐Bi in 1 m Na_2_CO_3_. Reproduced with permission.^101^ Copyright 2014, American Chemical Society. Schematics of the c) planar and d) 3D nanostructured cocatalysts deposited onto a photoelectrode. The PEC *J–V* curves of a photoelectrode with e) planar and f) 3D‐structured cocatalysts. *A*_cat_ indicates the proportion of the metal cocatalysts on the whole surface of a photoelectrode. *A*_3D_ is the increased surface area of the 3D catalyst structure with the given *A*_cat_. η_half‑STH_ is the half cell solar‐to‐hydrogen efficiency. As for traditional planar cocatalysts, an optimum *A*_cat_ exists for the maximum PEC performance. By contrast, a photoelectrode with the 3D structured cocatalysts can achieve the ultimate PEC performance of the semiconductor and cocatalyst materials. Reproduced with permission.^245^ Copyright 2017, American Chemical Society.

Cocatalysts can be divided into isolated patches or overlayers. The catalytic activities of these cocatalysts largely depend on their optically transparent property, thickness, size, morphology, footprint area on the surface of semiconductors, etc. As for cocatalyst overlayers, the catalytic activities can be determined by their thickness. Eftekharinia et al.^108^ reported the deposition of Co–Pi layer onto hematite and found the overlayer had an optimized thickness at a deposition time of 5 min, and when the layer became much thicker, a negative effect will occur due to the difficult mass transfer inside it. It is noteworthy that the intrinsic properties of different materials will affect the correlation between the thickness of cocatalysts and the PEC‐OER performance. Chemelewski et al.^101^ reported that for very thin films, α‐FeOOH has nearly the same activity as Co–Bi, while for thicker films Co–Bi performs better than α‐FeOOH (**Figure** **22**a) because of the solution penetration of Co–Bi which α‐FeOOH does not possess. This ion permeation property of Co–Bi produces more active sites in Co–Bi films with thickness increasing which is beneficial for better PEC performance, though it also brings about corrosion and reduces the stability of photoanodes simultaneously. By contrast, despite that thicker film of α‐FeOOH means less activity, it also hints that it can better protect unstable photoanodes and ensure the photoanode to maintain a good performance in a large range of pH conditions, as can be seen in Figure 22b. Another dimension that contributes to the difference of catalytic performance of cocatalysts is crystallinity. Xu et al.^178^ reported the Ti‐Fe_2_O_3_ photoanodes modified with CoOOH of different crystallinity (Co‐L, Co‐M, Co‐H) for PEC water oxidation. CoOOH with low crystallinity is highly ion‐permeable, leading to an intimate interaction between semiconductor and electrolyte rather than CoOOH and semiconductor; CoOOH with high crystallinity is close to ion‐impermeable, showing a low catalytic activity because of the sluggish charge transfer; While CoOOH with moderate crystallinity is partially electrolyte‐screened which is beneficial to form an ideal band bending and lower overpotentials of water oxidation. Actually, the size of cocatalytic particles is also a critical factor affecting cocatalyst activity in PEC. For metal particles, larger cocatalyst particles are more effective for performance enhancement, since larger particles can assist them in extracting holes more efficiently and providing less charge recombination centers at the cocatalyst/particle interface if they are in the size range of the depletion width, which has already been verified by the solid‐state theory.^138^ In addition, Oh et al.^245^ also revealed that the catalytic activities of 2D planes (Figure 22c) differ significantly with that of 3D nanostructures (Figure 22d). And Figure 22e presents that in terms of 2D planar cocatalyst, the current density declines with footprint area increasing. They explained that although increasing footprints could provide more reaction sites, reduce overpotential, and enhance PEC‐OER rate, the worse optical shading effect due to the increasing cocatalyst footprint decreases the photocurrent and limits its performance. With regard to the structured cocatalysts, with increased reaction sites, as can be seen in Figure 22f, the electrochemical overpotential can be significantly reduced as the footprint increases though a limited current density maintains. Besides, as the surface area increases, the PEC efficiency also increases along with it. They attributed the result to the large surface area and the lower light absorption from the surface area of the cocatalysts.

In total, the catalytic performance of cocatalysts is complex and synergistically affected by many dimensions such as the aforementioned ones rather than just linearly related with a certain parameter. Therefore, to optimize the capability of cocatalysts for better facilitating solar water oxidation, many influences need to be taken into consideration and some trade‐offs are necessary to be made.

## 4 Conclusions and Perspective

The work of Fujishima and Honda on semiconductor electrodes in 1972 is a historical turning point in the energy field, which opened up a new gateway of solar applications and laid the foundation of photo‐electrochemical water splitting technology. The PEC water splitting system can not only eliminate expensive investment of electrolyzers, but also has the potential to further reduce cost through enhancing the solar‐energy‐conversion efficiency. And up till now, many advances have been achieved due to the unremitting endeavor of researchers. With the development of PEC water splitting, the researchers are no longer satisfied with the PEC water splitting study itself. In the recent years, the integration of PEC systems with photovoltaic (PV) cells and the study of bifunctional PEC systems have become the new favorites of some researchers. In a PEC–PV integration system, PV cells can play a role of bias supplier, which is conducive to reducing initial capital and operation cost. A WO_3_/BiVO_4_ photoanode with a GaAs/InGaAsP PV cell as the bias provider is constructed by Pihosh et al. for water splitting, reaching a total STH efficiency of up to 8.1%.^269^ PEC cells can not only convert solar energy to chemical fuels, some of them can also degrade pollutants simultaneously. Park et al. demonstrated the design of multilayered BiO*_x_*‐TiO_2_ electrodes, which were utilized for phenol degradation as well as H_2_ production simultaneously.^270^

However, all these efforts still cannot support the PEC water splitting systems for further commercialization currently. US Department of Energy once set a benchmark conversion efficiency of 10% for commercialization application.^271^ However, the efficiency for most of the PEC water splitting systems at present is still stuck below this benchmark. Even though a tiny minority of them can reach this benchmark, either the stability^272^ cannot meet the demand of practical usage or the cost^273^ is still significantly higher than the target of $ 2–4 per kg for H_2_ production^274^ yet. Except that, other considerations such as accessibility, environmental friendliness, renewability, and simplification still remain to be realized. In total, the realization of the commercial application of PEC water splitting in the future will be a long‐term course full of challenges and difficulties. However, for now, the most imperative mission is still to significantly boost the performance and stability of PEC water splitting, especially PEC water oxidation via effective strategies.

With an initial purpose of introducing the concept of PEC water splitting, especially the PEC water oxidation half reaction, demonstrating the important status of the water oxidation cocatalysts in the PEC water splitting system, and providing tutorial guidance for researchers to conduct relative researches, this review systematically and detailedly provides a basic framework of PEC water oxidation half reaction, including the basic principles of water splitting, the major modules to establish a PEC water oxidation system, essential parameters extensively used to evaluate their performance of PEC cells. Besides, this review also intensively introduces the mechanisms and the categories of promising cocatalyst materials (**Table** **4**) that have been widely used and newly developed in recent years such as metal oxides or (oxy)hydroxides, LDHs, transition metal phosphides (TMPs), metal‐free materials, molecular materials, etc. These cocatalysts all emerge their own advantages such as earth‐abundance, stability, as well as high catalytic activity when loading onto the surface of photoanodes. It is also concluded that these cocatalysts help PEC water oxidation by increasing band bending, serving as the hole trap to collect holes from semiconductors, or even acting as the protecting layer to prevent from photocorrosion. However, from another facet, some cocatalysts cannot match the bandgap position of specific photoanodes. Except that, cocatalyst application may also induce optical shield effect and added recombination sites, neither of which are favorable for PEC water oxidation. Hence, these intractable problems of cocatalysts still need to be coped with in the future.

**Table 4:** The summary of common cocatalysts and their PEC OER performance

Cocatalysts		Photoanodes	Deposition method	Morphology	Photocurrent density [mA cm^−2^]	Onset potential	Conditions	Ref.
Metal phosphates	Co–Pi	Fe_2_O_3_/Co–Pi	Electron beam evaporation	Thin films	1.5 at 1.5 *V*_RHE_	0.9 *V*_RHE_	1 m NaOH (pH = 13); under AM 1.5G (100 mW cm^−2^)	108
	Co–Pi	Fe_2_O_3_/CdS/Co–Pi	Photo‐assisted deposition	Nanoparticles	3.29 at 0.7 *V*_RHE_	0.4 *V*_RHE_	1.0 m NaOH + 0.1 m Na_2_S (pH = 13.31); under AM 1.5G (100 mW cm^−2^)	50
	Co–Pi	BiVO_4_/Co–Pi	Photo‐electrodeposition	Films	3 at 1.23 *V*_RHE_	0.4 *V*_RHE_	0.1 m NaPi buffer (Ph ≈ 7); under AM 1.5G (100 mW cm^−2^)	110
	NiFe–Pi	NiFe–Pi/α‐Fe_2_O_3_	Facile dip‐coating and annealing	Nanoparticles	1.2 at 1.23 *V*_RHE_	0.74 *V*_RHE_	0.1 m KOH (pH = 12.6) under AM 1.5G (100 mW cm^−2^)	117
Metal phosphides	CoP	Mo:BiVO_4_/CoP	Drop casting	Nanoparticles	3.0 at 1.23 *V*_RHE_	0.3 *V*_RHE_	0.5 m KPi buffer (pH ≈ 7) under AM 1.5G (100 mW cm^−2^)	124
	FeMnP	TiO_2_/FeMnP	Metal–organic chemical vapor deposition	Film	1.8 at 1.23 *V*_RHE_	≈0 *V*_RHE_	0.5 m NaOH under AM 1.5G (100 mW cm^−2^)	118
Metal (oxy)hydroxides	Ni(OH)_2_	BiVO_4_/Ni(OH)_2_	Chemical bath deposition	Ultrathin layer	≈0.75 at 1.23 *V*_RHE_	≈0 *V*_RHE_	NaBi (pH = 10) buffer; under AM 1.5G (100 mW cm^−2^)	163
	NiOOH	(Sn,Zr) α‐Fe_2_O_3_/NiOOH	Photo‐assisted electrodeposition	Rough films	1.64 at 1.23 *V*_RHE_	0.58 *V*_RHE_	1 m NaOH (pH = 13.6), under AM 1.5G (100 mW cm^−2^)	169
	FeOOH	FeOOH/BiVO_4_	Facile solution impregnation	Nanolayer (2 nm)	4.3 at 1.23 *V*_RHE_	0.5 *V*_RHE_	0.2 m Na_2_SO_4_; under AM 1.5G (100 mW cm^−2^)	73
	Ni:FeOOH	Ni:FeOOH/BiVO_4_	Hydrothermal process	Films	1.6 at 0.4 *V*_Ag/AgCl_	−0.2 *V*_Ag/AgCl_	0.1 m Tris‐PBS 5.0; under AM 1.5G (100 mW cm^−2^)	159
	CoOOH	CoO(OH)/Ti‐Fe_2_O_3_	Electrochemical deposition	Crystalline layers	1.8 at 1.23 *V*_RHE_	0.7 *V*_RHE_	1 m NaOH (pH = 13.6) under AM 1.5G (100 mW cm^−2^)	178
Metal oxides	MOFsa)‐derived Co_3_O_4_	MOFs‐derived Co_3_O_4_/TiO_2_/Si	Electrodeposition/hydrothermal method	Hierarchical structure	2.71 at 1.23 *V*_RHE_	0.4 *V*_RHE_	1 m NaOH; under AM 1.5G (100 mW cm^−2^)	141
	NiO*_x_*	p^+^n^−^InP|NiO*_x_*	Sputter deposition	Films	17.1–17.9 at 1.23 *V*_RHE_	0.85–0.87 *V*_RHE_	1.0 m KOH; under AM 1.5G (100 mW cm^−2^)	143
	NiMoO_4_	TiO_2_/Si/NiMoO_4_	Hydrothermal method	Nanosheets	1.4 × 10^−2^ at 1.23 *V*_RHE_	0.85 *V*_RHE_	0.25 m KOH; under AM 1.5G (100 mW cm^−2^)	149
	FeNiO*_x_*	Fe_2_TiO_5_/FeNiO*_x_*	Photo‐electrochemical deposition	Films	0.93 at 1.23 *V*_RHE_	≈0.6 *V*_RHE_	1 m NaOH; under AM 1.5G (100 mW cm^−2^)	265
Layered double hydroxides	ZnFe‐LDH	TiO_2_/ZnFe‐LDH	Photo‐electrochemical deposition	Nanoplates	1.51 at 1.23 *V*_RHE_	0.4 *V*_RHE_	0.5 m Na_2_SO_4_; under AM 1.5G (100 mW cm^−2^)	74
	NiCoAl‐LDH	α‐Fe_2_O_3_/NiCoAl‐LDH	In situ growth	Films	2.56 at 1.23 *V*_RHE_	0.55 *V*_RHE_	0.5 m K‐Pi, (pH = 7); under AM 1.5G (100 mW cm^−2^)	48
Metal‐based boron compounds	Ni–Bi	H‐BiVO_4‐_ *_x_*/Ni–Bi	Photodeposition	Films	3.34 at 1.23 *V*_RHE_	0.02–0.1 *V*_RHE_	0.1 m KBi (pH = 9); under AM 1.5G (100 mW cm^−2^)	212
	NiB	BiVO_4_/NiB	Drop‐casting	Nanoparticles	3.47 at 1.23 *V*_RHE_	0.25 *V*_RHE_	0.5 m KBi (pH = 9.20); under AM 1.5G (100 mW cm^−2^)	216
Metal‐free materials	N‐CDsb)	α‐Fe_2_O_3_/Ti/N‐CDs	Facile “top down” method	Nano dots	0.41 at 1.23 *V*_RHE_	0.79 *V*_RHE_	1 m KOH (pH = 13.6); under AM 1.5G (100 mW cm^−2^)	229
	C_3_N_4_	BiVO_4_/g‐C_3_N_4_	Ethylene glycol dispersion and impregnation	Ultrathin nanosheets	3.12 at 1.23 *V*_RHE_	0.4 *V*_RHE_	0.1 m Na_2_SO_4_; visible‐light (λ > 420 nm)	217
Metal atoms	NiFe alloy IOsc)	p^+^n‐Si/SiO_2_/NiFe IOs	Electrodeposition	3D nanoporous network	31.5 at 1.23 *V*_RHE_	0.94 *V*_RHE_	1 m KOH; under AM 1.5G (100 mW cm^−2^)	245
Molecules	Ir WOCd)	Fe_2_O_3_/Ir WOC	Anodic photo‐electrodeposition	Amorphous monolayer	≈0.9 at 1.23 *V*_RHE_	0.6 *V*_RHE_	0.1 m KNO_3_+HNO_3_ (pH = 1.01); under AM 1.5G (100 mW cm^−2^)	266
Dual cocatalysts	IrO_2_/RuO_2_	IrO_2_/RuO_2_/hematite	Electrodeposition	Films	≈1.52 at 1.23 *V*_RHE_	0.5 *V*_RHE_	1 m KOH (pH = 13.6) under AM 1.5G (100 mW cm^−2^)	267
	FeOOH/Au	Fe_2_O_3_/FeOOH/Au	Facile “top down” method	FeOOH: Films/Au: nanoparticles	3.2 at 1.23 *V*_RHE_	0.6 *V*_RHE_	1 m KOH; under AM 1.5G (100 mW cm^−2^)	173
	Co cubane/Ir complex	Ni(OH)*_x_*/Co cubane/Ir complex/Fhe)/TiO*_x_*/Ta_2_N_5_	Facile “top down” method	Particles	12.1 at 1.23 *V*_RHE_	0.65 *V*_RHE_	1 m NaOH; under AM 1.5G (100 mW cm^−2^)	79
	CdTe QDsf)/LDH	CdTe QDs/LDH/BiVO_4_			2.23 at 1.23 *V*_RHE_	0.3 *V*_RHE_	0.1 m phosphate buffer (pH = 7); under AM 1.5G (100 mW cm^−2^)	268
Others	(MOF) MIL‐100(Fe)	Fe/W codoped BiVO_4_/(MOF) MIL‐100(Fe)	Spin‐coating	Nanoparticles	2.76 at 0.8 *V*_SCE_	0 *V*_SCE_	0.1 m Na_2_SO_4_; under AM 1.5G (100 mW cm^−2^)	262
	CoFe–PBg)	CoFe‐PB/BiVO_4_	Sequentially dipping	Amorphous particles	≈1.17 at 1.23 *V*_RHE_	0.3 *V*_RHE_	0.1 m KPi buffer (pH ≈ 7); under AM 1.5G (100 mW cm^−2^)	263

^a)^ MOF: metal–organic framework

^b)^ N‐CDs: N‐Doped carbon dots

^c)^ IOs: inverse opals

^d)^ WOC: water oxygen catalysts

^e)^ Fh: ferrhydrite

^f)^ QDs:quantum dots

^g)^ CoFe–PB: CoFe Prussian blue‐type electrocatalysts

In fact, despite that the research of cocatalysts has continued for many decades and some tolerable progress has been made, few significant breakthroughs are achieved to assist the study of cocatalyst in making a giant leap currently. Accordingly, there is a long way to go forward in order to gain cocatalysts with much higher performance and ultimately achieve commercial water splitting. It is anticipated that in the near future, many works based on the cocatalysts applied in PEC water oxidation will focus on the following aspects:

### 4.1 In‐Depth Interpretation of Mechanisms

The mechanisms how cocatalysts work have not yet been stated clearly. Some of the explanations are based on hypothesis and the results of characterization, which actually do not have enough persuasion. Besides, some paradoxes still exist and in certain papers which focus on the synergies of cocatalysts, the clarifications of their effects are still superficial. Thus, further rationally designed experiment verifications, through characterizations, maintain to be conducted in order to provide much more powerful evidences and specific explanations. In addition, theoretical calculations, especially density functional theory study, are of equal importance. It can make up the defects of experimental measurements and plays an important role in validation^275^ and prediction.^276^ Hence, these strategies are recognized as a rather powerful ancillary tool to better understand the results of experiments, investigate the underlying mechanism how cocatalysts work, and provide practical guidance for designing high‐performance cocatalyst‐assisted PEC water oxidation system.

### 4.2 The Exploration of New Efficient and Sustainable Catalyst Materials

Novel materials like metal‐free materials, molecular materials, or MOFs are all worthy to be explored for expanding the family of cocatalysts and offering more options. As mentioned in introduction, most electrocatalysts can be used as PEC cocatalysts. Thus, the state‐of‐art electrocatalysts can probably provide some inspirations for us in exploring novel PEC cocatalysts.

### 4.3 The Further Regulation and Optimization of Cocatalysts

Hitherto, the properties of cocatalysts are still far from satisfying the requirement of practical usage. There is a large space for us to further improve the performance of cocatalysts. Feasible methods include manufacturing defects or vacancies, nanostructuring, rational selection of conductive substrates, improved deposition method, morphology regulation, interfacial modulation, structure control, size adjustment, cocatalyst synergies, etc.

### 4.4 Coupling the Means of Cocatalyst Introduction with Others to Synergistically Promote the PEC‐OER Performance

For example, heterojunctions, dye sensitization, ion doping, surface defects, plasmon resonance technology, and piezoelectric technology, etc., can all be considered. As the PEC water oxidation process is relatively complex, evolving multielectron transfer and multiple reaction steps, it is hard to reach a more outstanding catalytic efficiency and higher stability far beyond current average level and realize industrialization via depositing cocatalyst alone. Hence, it is indispensable to combine cocatalysts loading and other methods for much higher performance.

### 4.5 The Design of Multifunctional Catalysts

Fabricating cocatalysts to act as both OER and HER excellent cocatalysts so as to realize ultimate overall water splitting with the same cocatalyst is one of the ideals of PEC application. Due to the incompatible pH conditions of traditional HER cocatalysts (normally work under pH < 7) and OER cocatalysts (usually conducted under pH > 7), it becomes difficult to assemble an overall water splitting system with anode and cathode coupled with HER and OER cocatalysts respectively at the same time, and the performance is often below our expectation. Thus, looking for some bifunctional cocatalysts to facilitate overall water splitting is of great importance in the future investigation of PEC overall water splitting system.^277^

All in all, the PEC water oxidation plays an indispensable role in sufficient solar light utilization and oxygen evolution. Therefore, the research of PEC water oxidation cocatalysts is of great value and deserves to be further investigated. It is expected that this review could not only deepen the comprehension of PEC water splitting and the role of water oxidation cocatalysts for artificial PEC synthesis but also open up new inspirations and feasibilities for cocatalyst applications.

## Conflict of Interest

The authors declare no conflict of interest.
